# Design of Novel 4-Aminobenzofuroxans and Evaluation of Their Antimicrobial and Anticancer Activity

**DOI:** 10.3390/ijms21218292

**Published:** 2020-11-05

**Authors:** Elena Chugunova, Almir Gazizov, Marina Sazykina, Nurgali Akylbekov, Anastasiya Gildebrant, Ivan Sazykin, Alexander Burilov, Nurbol Appazov, Shorena Karchava, Maria Klimova, Alexandra Voloshina, Anastasia Sapunova, Syumbelya Gumerova, Ayrat Khamatgalimov, Tatiana Gerasimova, Alexey Dobrynin, Olga Gogoleva, Vladimir Gorshkov

**Affiliations:** 1 Arbuzov Institute of Organic and Physical Chemistry, FRC Kazan Scientific Center, Russian Academy of Sciences, Kazan, Tatarstan 420088, Russia; burilov@iopc.ru (A.B.); sobaka-1968@mail.ru (A.V.); microbi@iopc.ru (A.S.); syumbelya07@mail.ru (S.G.); ayrat_kh@iopc.ru (A.K.); tatyanagr@gmail.com (T.G.); aldo@iopc.ru (A.D.); 2 Laboratory of Plant Infectious Diseases, FRC Kazan Scientific Center of Russian Academy of Sciences, Kazan, Tatarstan 420111, Russia; gogolewaoa@yandex.ru (O.G.); gvy84@mail.ru (V.G.); 3 Southern Federal University, Rostov-on-Don 344090, Russia; submarinas@list.ru (M.S.); siniamida@gmail.com (A.G.); zebra-sis@yandex.ru (I.S.); skarchava@sfedu.ru (S.K.); mzhuravleva@sfedu.ru (M.K.); 4 Laboratory of Engineering Profile “Physical and Chemical Methods of Analysis”, Korkyt Ata Kyzylorda University, Kyzylorda 120014, Kazakhstan; nurasar.82@mail.ru; 5 I. Zhakaev Kazakh Scientific Research Institute of Rice Growing, Kyzylorda 120008, Kazakhstan

**Keywords:** benzofuroxan, anti-cancer activity, apoptosis, bacterial biofilm, fungistatic activity, quantum chemical calculations

## Abstract

A series of novel 4-aminobenzofuroxan derivatives containing aromatic/aliphatic amines fragments was achieved via aromatic nucleophilic substitution reaction of 4,6-dichloro-5-nitrobenzofuroxan. The quantum chemistry calculations were performed to identify the factors affecting the regioselectivity of the reaction. The formation of 4-substituted isomer is favored both by its greater stability and the lower activation barrier. Antimicrobial activity of the obtained compounds has been evaluated and some of them were found to suppress effectively bacterial biofilm growth. Fungistatic activity of 4-aminobenzofuroxans were tested on two genetically distinct isolates of *M. nivale*. The effect of some benzofuroxan derivatives is likely to be more universal against different varieties of *M. nivale* compared with benzimidazole and carbendazim. Additionally, their anti-cancer activity in vitro has been tested. 4-aminofuroxans possessing aniline moiety showed a high selectivity towards MCF-7 and M-HeLa tumor cell lines. Moreover, they exhibit a significantly lower toxicity towards normal liver cells compared to Doxorubicin and Tamoxifen. Thus, benzofuroxans containing aromatic amines fragments in their structure are promising candidates for further development both as anti-cancer and anti-microbial agents.

## 1. Introduction

Heterocyclic *N*-oxides hold a key position in the chemistry of organic compounds due to their usefulness as versatile synthetic intermediates and their biological importance. This motif has been successfully employed in a number of recent drug development investigations. There are numerous examples of antibacterial, anti-tuberculosis, antineoplastic, and other drugs derived from heterocyclic *N*-oxides [1,2]. Among them, benzofuroxans represent the emerging class of compounds, which exhibit various biological activities [3,4,5,6,7,8,9]. Unsubstituted benzofuroxan and its chlorinated derivatives have been proposed as bactericides and fungicides for the treatment of seed peas, beans and potatoes, tetrabromotetrahydrobenzofuroxan has been proposed for agricultural use as an antifungal agent [10]. Notably, halogen-containing benzofuroxans are much more potent compared to their unsubstituted counterparts [11]. Additionally, the possibility of aromatic nucleophilic substitution of halogen atoms makes them promising candidates for further design of novel bioactive molecules.

Molecular modification is a rather promising strategy in the development of drug analogues with better bioavailability, higher biological activity, and less toxicity. Special attention is currently paid to the “hybrid” compound containing benzofuroxan and another biologically active subunit in one molecule [3,12,13]. Evaluation in vitro and in vivo of levels of prostaglandin E2 and thromboxane B2 decrease in the blood plasma samples of a compound obtained by binding of diclofenac and benzofuroxan fragments showed that after adding of benzofuroxan scaffold, this compound retains the pharmacological characteristics of Diclofenac, including anti-inflammatory activity, without the deleterious gastrointestinal effects which is normally present in the prototype [14]. The creation of novel “hybrid” compounds based on nitrobenzofuroxans is very important due to the high fungicidal and bactericidal activity inherent in their structure, the absence of toxicity and genotoxicity, as well as the possible presence of various protective biological effects.

In this work, we chose aromatic amines as the second fragment for “hybrid” compounds based on benzofuroxans. Aromatic amines are widely used as starting materials in pharmacology, fur and textile industries. They also serve as useful intermediates for the synthesis of numerous organic compounds [15,16], including azo dyes, pigments, and as antioxidants in the manufacture of most rubber products [17]. However, most of them are highly toxic to living organisms, many of these aromatic compounds are carcinogenic and mutagenic for humans [18]. Notably, the literature data evidences that the introduction of a NO-donor benzofuroxan fragment into the molecule may result in its increased bioavailability and the pharmacological activity and reduced toxicity compared to the parent compound [14,19,20]. An additional advantage of using aromatic amines as the second fragment in the synthesis of “hybrid” compounds is their low cost and availability.

In order to show the importance of introducing namely fragments of aromatic amines into the structure of benzofuroxans, we also synthesized a number of compounds containing fragments of aliphatic amines and studied the anticancer and antimicrobial activities of both series of compounds. Future, using the example of the reaction 4,6-dichloro-5-nitrobenzofuroxan with aniline, we first explained the regioselectivity of the substitution of the chlorine atom in the 4 position with help of quantum chemical studies.

## 2. Results and Discussion

### 2.1. Chemistry

#### 2.1.1. Synthesis

**Scheme 1:**
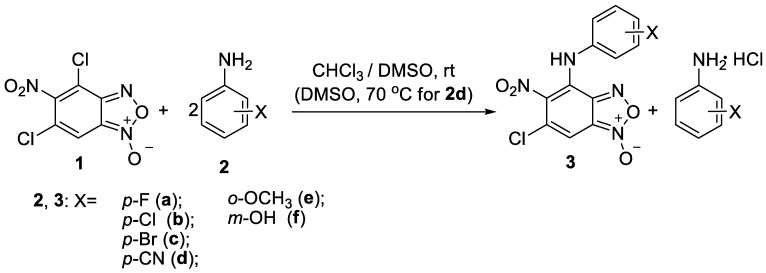
Reaction of 4,6-dichloro-5-nitrobenzofuroxan **1** with aromatic amines.

Key compounds were obtained via aromatic nucleophilic substitution reaction of 4,6-dichloro-5-nitrobenzofuroxan **1** using the aromatic amines **2a-e** as nucleophiles (Scheme 1).

As in the previously illustrated examples [12,13], the obtained results indicate that the reactivity of two chlorine atoms in benzofuroxan **1** towards nucleophilic substitution differs significantly. The substitution occurred in the position 4 of the benzofuroxan **1** solely regardless of the reactants ratio, reaction time and the temperature. Thus, the reaction with all aromatic amines used afforded monosubstituted products **3a-f** as the only products, even in the presence of great excess of the amine.

It should be noted that the reaction time depends on the nature of the substituent in the anilines **2**. In the case of the presence of electron-donating substituents the reaction finishes in 1–2 h at the room temperature. Vice versa, in the case of the presence of electron-withdrawing substituents the harsher conditions are required. For example, the reaction with *p*-aminobenzonitrile proceeds only upon heating the reagents at 70 °C in DMSO for 2 h. These observations are in agreement with the lower nucleophilicity of anilines possessing electron-withdrawing substituents.

**Scheme 2:**
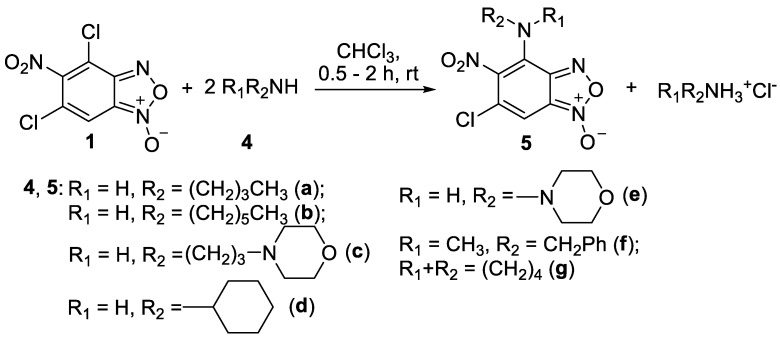
Reaction of 4,6-dichloro-5-nitrobenzofuroxan **1** with aliphatic amines.

In addition, to further compare the biological activity of the two series of compounds we have synthesized a series of benzofuroxan derivatives containing fragments of aliphatic amines. The reaction proceeded similarly and resulted in 4-aminobenzofuroxans **5a-e** (Scheme 2). The secondary aliphatic amines, both cyclic and acyclic, also behaved well providing compounds **5f**,**g**, [acyclic: methylbenzylamine **4f** and cyclic: pyrrolidine **4g**] (Scheme 2). Despite the greater nucleophilicity of aliphatic amines compared to anilines, no substitution occurred at the position 6 of benzofuroxan **1**.

**Figure 1:**
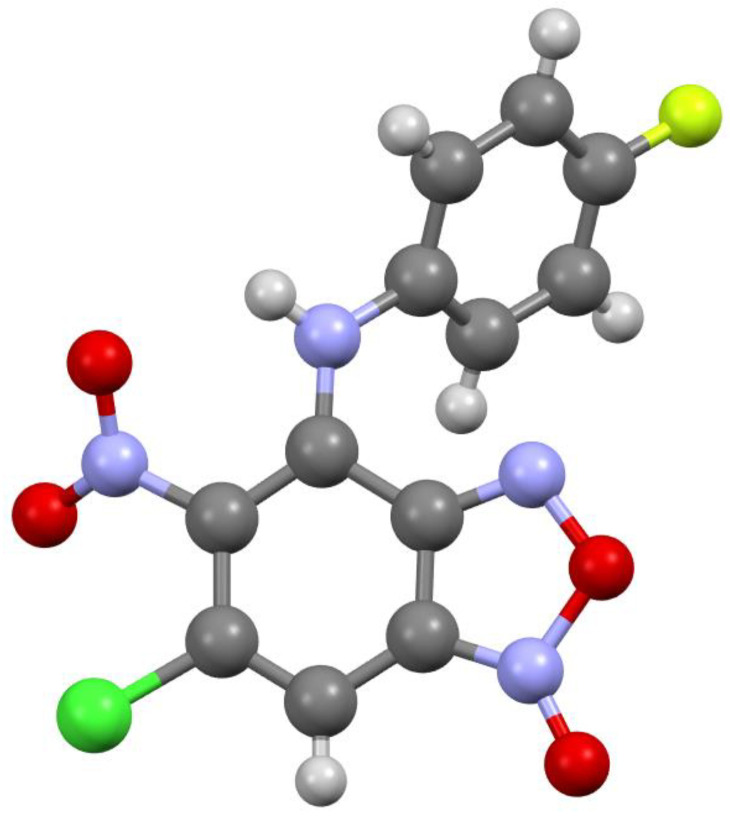
Molecular structure of compound **3a**.

**Figure 2:**
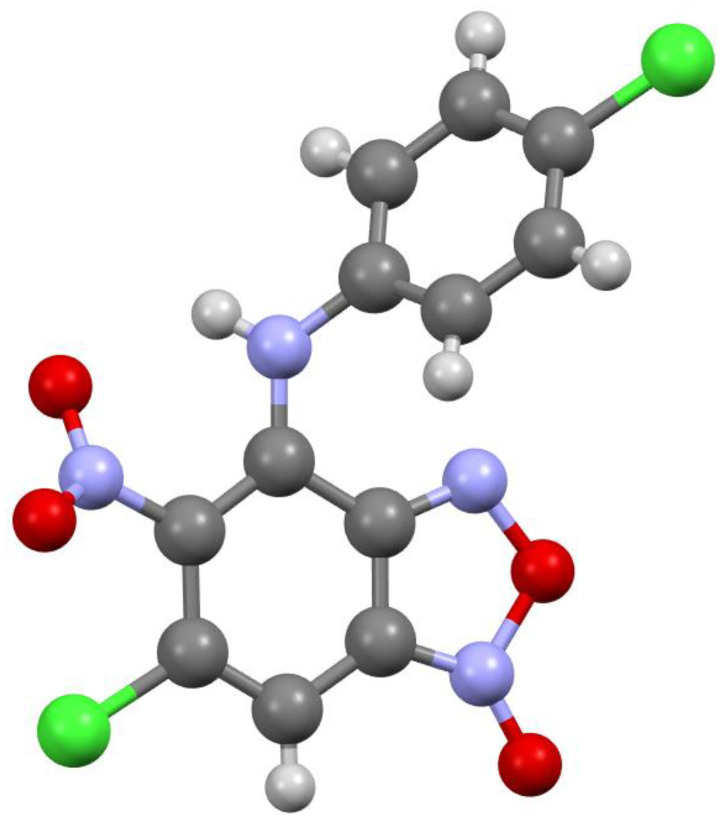
Molecular structure of compound **3b**.

**Figure 3:**
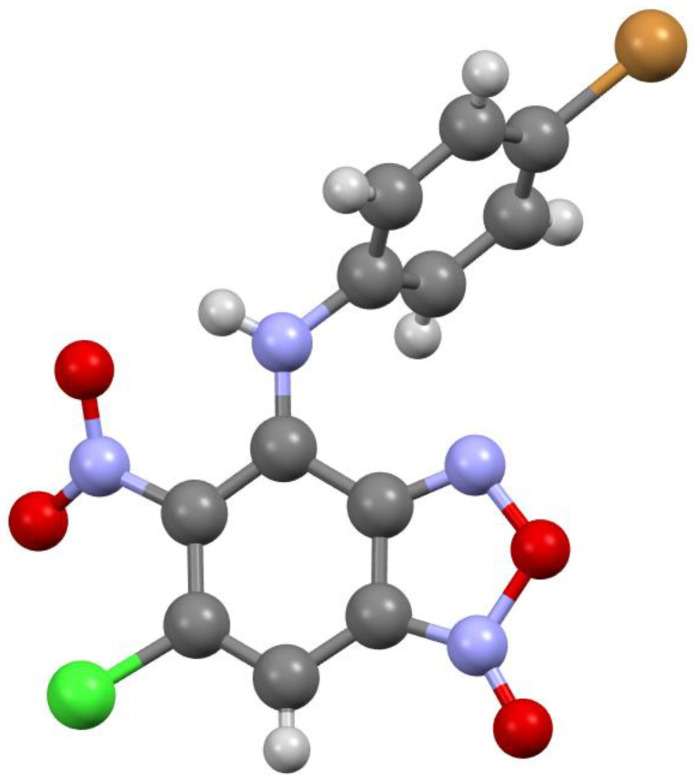
Molecular structure of compound **3c**.

**Figure 4:**
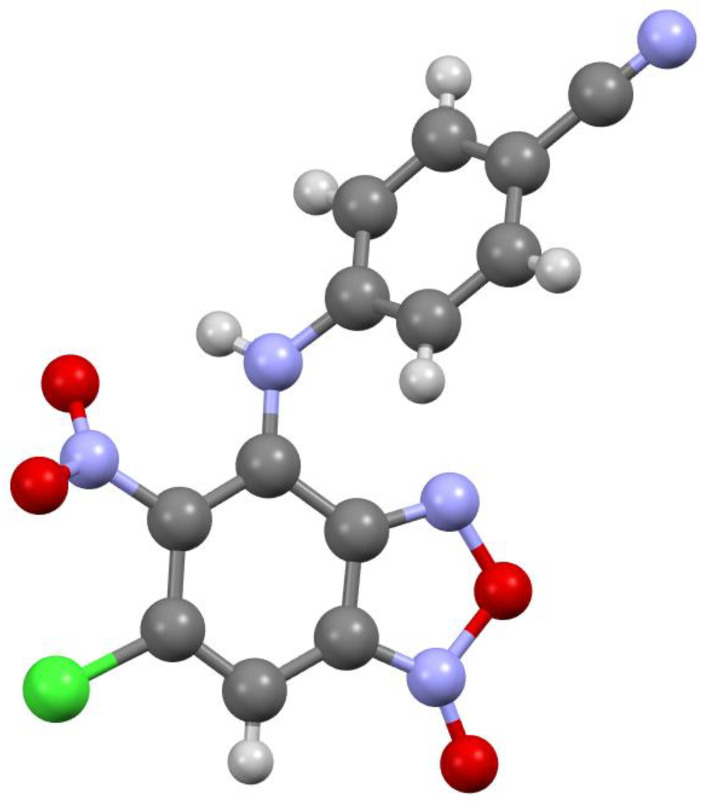
Molecular structure of compound **3d.**

The key point in the elucidation of structures of obtained compounds **3a-f** and **5a-g** was the exact determination of their substitution site. This was achieved by X-ray experiments for the compounds **3a-d** (see below, Figure 1, Figure 2, Figure 3 and Figure 4). The substitution site of the compound **5c** was determined through careful analysis of the ^13^C NMR spectrum and its side-by-side comparison with the ^13^C NMR spectra of compounds **3a-d**. The substitution site of other compounds was determined similarly using previously described 6-chloro-4-((2-(dimethylamino)ethyl)amino)-5-nitrobenzofuroxan [21] as reference.

According to X-ray data, compounds **3a**, **3b** and **3d** crystallize in the monoclinic space group *P2_1_/c*. Compounds **3a** and **3b** are isostructural. Compound **3c** crystallizes in the orthorhombic space group *Pbca* (Figure 1, Figure 2, Figure 3 and Figure 4). Benzofuroxan’s bicyclic fragments are planar, and substituted phenyl rings deviate from the plane of the bicycle. Moreover, substituted phenyls of compounds **3a** and **3c** deviate from the plane of the bicycle on one side (torsion angles 33.14 and 17.44°, respectively), but in compounds **3b** and **3d** deviates on the other side (torsion angles 32.25 and 35.71°, respectively). It should also be noted that in the same pairs of compounds **3a**/**3c** and **3b**/**3d**, the nitro groups at aromatic rings deviate in different directions relative to the ring: in pair of the **3a**/**3c** the torsion angles are 16.9 and 27.63°, respectively, in the pair of **3b**/**3d** the torsion angles are 37.97 and 43.39°, respectively.

#### 2.1.2. Quantum Chemical Calculations

**Scheme 3:**
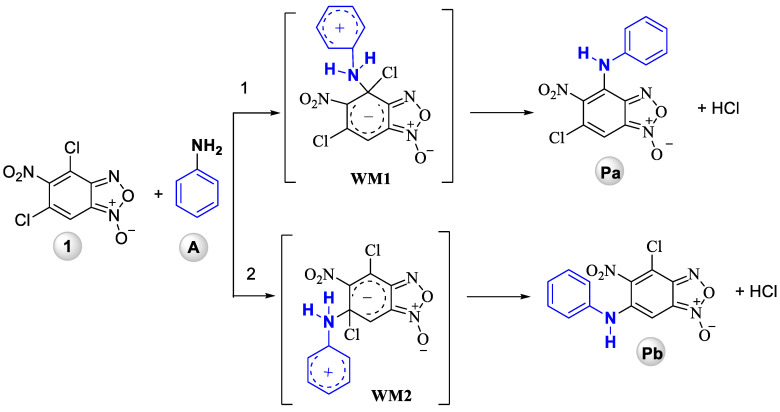
Two possible ways for reaction of 4,6-dichloro-5-nitrobenzofuroxan **1** with aniline (**A**).

Although, aromatic nucleophilic substitution (S_N_Ar) reactions of 4,6-dichloro-5-nitrobenzofuroxan and different amines has been reported previously [19,22] we have never tried to explain the reason for such an unusual phenomenon. The regioselectivity of this process becomes the major issue when a multiple functional groups are present in the molecule and more than one position can be substituted. The typical examples are reactions of 4,6-dinitro-5,7-dichlorobenzofuroxan, in which either of the chlorine atoms may be substituted [23] or the S_N_Ar reactions of polyhalogenated benzofused aza-heterocycles [24,25]. Similarly, the amination of pentafluoronitrobenzene leads to the mixture of *p*-, *o*- and *p*,*o*-, *o*,*o*-substituted products [26]. Thus, controlling the regioselectivity of the substitution in such substrates is a challenging task in the practically useful molecules design. Quantum chemistry methods have proved to be a valuable tool for the prediction or explanation of regioselectivity of S_N_Ar reactions. Various substrates have been studied, including polyhalogenated benzenes [27,28,29], perfluoronaphthlanes [30], polyhalogenoheterocycles [31,32,33] and their number continues to grow. Despite this, benzofuroxans received a little attention and this area still remains rather unexplored. To shed light on the peculiarities of interaction of 4,6-dichloro-5-nitrobenzofuroxan **1** with aniline (**A**) quantum-chemical study of this reaction has been carried out. Two possible ways have been considered: substitution of Cl atom (i) in 4 (**Pa**) and (ii) in 6 (**Pb**) positions with formation of Wheland–Meisenheimer zwitterionic intermediates (**WM** on Scheme 3).

According to quantum chemical calculations results, the reaction of 5-nitro-4,6-dichlorobenzofuroxan **1** with aniline **A** is exothermic (thermal effect is 11.1 and 3.7 kcal/mol, respectively, for the two expected reaction pathways, see Table 1) with **Pa** being thermodynamically more stable compared to **Pb**, higher HOMO-LUMO gap of **Pa** suggests also its higher kinetical stability (Table 2).

Thus, quantum chemical modeling shows that the formation of **Pa** (6-chloro-5-nitro-4-(phenylamino)benzofuroxan) is preferable compared to **Pb** (4-chloro-5-nitro-6-(phenylamino)benzofuroxan) that is proved by thermodynamic stability of the reaction product, thermal effect of the reaction and lower activation barrier of **Pa** (6-chloro-5-nitro-4-(phenylamino)benzofuroxan) formation (for details, see Supplementary Materials, Figures S1–S3, Table S1). Thus, the quantum chemistry calculations support fully the experimental data obtained earlier and in this investigation and evidences the greater reactivity of the chlorine atom in the 4th position of the 4,6-dichloro-5-nitrobenzofuroxan **1**.

### 2.2. Biological Studies

#### 2.2.1. Anti-Biofilm Activity of Benzofuroxan Derivatives

Currently the screening of bioactive compounds involves a large number of analysis assays that allow assessing the potential of biological extracts or molecules. The assays can be performed at the whole animal, cell-based or molecular levels [34]. 

Anyway, the search for substances which may be used as therapeutics for diseases associated with bacteria, is of great interest. A large number of laboratories and companies around the world are occupied with the problem of search of the medicines possessing complex medical action, and, in particular, antibacterial activities [35,36,37,38,39].

Microbial infections have become an important clinical threat, with significant associated morbidity and mortality, which is mainly due to the development of microbial resistance to the existing antimicrobial agents. Therefore, methods for antimicrobial susceptibility testing and discovery of novel antimicrobial agents have been extensively used. All of the most important groups of antibiotics are in danger of losing their efficacy because of the increase in microbial resistance. For this reason, the discovery of new antimicrobials is one of the main objectives of medicine nowadays. One of the main strategies of bacteria survival in the organisms of the infected owners is formation of biofilm communities of microorganisms. This way of bacteria existence creates big problems in medical practice. In this regard, the search for and studying of substances which can suppress formation of biofilms and kill bacteria in biofilms is an extremely important and relevant problem of antimicrobial therapy.

Considering the abovesaid, it was of interest to define anti-biofilm activity in our substances.

The data obtained during the study of biofilm formation degree by the strain *Vibrio aquamarinus* DSM 26054 in the presence of the studied substances are shown in Table 3 (see Supplementary Materials, Figures S4–S14 for additional data).

Substances **3a**, **3c**, **3d**, **5e** are able to both stimulate and inhibit biofilms formation by the strain *V. aquamarinus* DSM 26054 depending on the concentration. Similar effects were shown in our earlier works for various 1-[(2-aminoethyl)sulfonyl]-2-arylpyrrolidines, 2-(pyrazolyl)pyrrolidines and pyrrolidine-1-carboxamides [40,41,42]. Such an oppositely directed dose-dependent effect is known for various groups of substances, including antibiotics, and is described in the literature as hormesis [43,44,45]. Such effects are of interest for further studies, but due to this substance **3a**, **3c**, **3d**, **5e** are not recommended to be used as pharmaceuticals capable of inhibiting the biofilm development of vibrios. 

Substances **3b**, **3e**, **3f, 5b**, **5f**, **5g** had an inhibitory effect on biofilm formation in a number of studied concentrations. Biofilm formation compared to control ranged from 5.78% to 94.48%. The maximum inhibitory effect was exerted by **3b** substance at the concentration of 10^−4^ M (biofilm formation was 5.78%). At the concentration of 10^−5^ M, the inhibitory effect was recorded when exposed to substances **3b** and **3e**—the intensity of biofilm formation compared to the control was 46.33% and 65.93%, respectively. At the concentration of 10^−6^ M, substances **3f, 5b** and **5g** demonstrated the ability to suppress the development of biofilms (the intensity of biofilm formation was 43.56, 88.43% and 79.81%, respectively). Substances **3b**, **3e**, **3f, 5b**, **5f**, **5g** showed anti-biofilm activity at the concentration of 10^−7^ M; the intensity of biofilm formation varied from 11.34 (**3f**) to 63.58% (**5f**). At the concentration of 10^−8^ M, substances **3b**, **3f**, **5b**, **5f**, and **5g** displayed a pronounced inhibitory effect—the intensity biofilms of biofilm formation ranged from 9.31% (**3f**) to 26.88% (**5g**). At the concentration of 10^−9^ M, substances **3b**, **5f** and **5g** effectively inhibited the formation of (the intensity of biofilm formation compared to the control was 29.56%, 25.67%, and 45.78%, respectively).

The tested compounds were also compared with a standard antibiotic Azithromycin (see Supplementary Materials for additional data). Azithromycin exhibited an insignificant biofilm suppression at a high concentration. Azithromycin suppressed the intensity of biofilm formation by *V. aquamarinus* DSM 26054 at the concentration of 10^−5^ M (Figure S14). The optical density was 81.5% of the control values.

Thus, 5 substances capable of suppressing the vibrios biofilm formation in low concentrations (10^−9^–10^−7^ M)—**3b**, **3e**, **3f**, **5b**, **5f**, **5g** were isolated.

#### 2.2.2. Fungistatic Activity of Benzofuroxans

Snow mold is a severe plant disease caused by psychrophilic and psichrotolerant fungi and fungi-like organisms [46]. These fungi damage mostly winter cereals as well as forage and turf grasses during autumn to spring period resulting in high crop losses. *Microdochium nivale (Fr.) Samuels and Hallet* is the most common reason for biotic winter crop damage [47,48,49]. Being a psychrotolerant fungus, *M. nivale* is able to cause disease not only at cool period, but also during the whole growing season leading to both snow mold as well as fusariosis-resemble diseases such as seedling blight, foot rot, leaf blight, and head blight [46]. *M. nivale* is a genetically diverse species represented by multiple genotypes [50,51]. The prevention of snow mold accounts for almost 50% of the yearly fungicide use on turfgrass in Canada [52]. Despite that, *M. nivale* remains common and serious pathogen, which is in particular related to the formation of fungicide-resistance [53].

**Figure 5:**
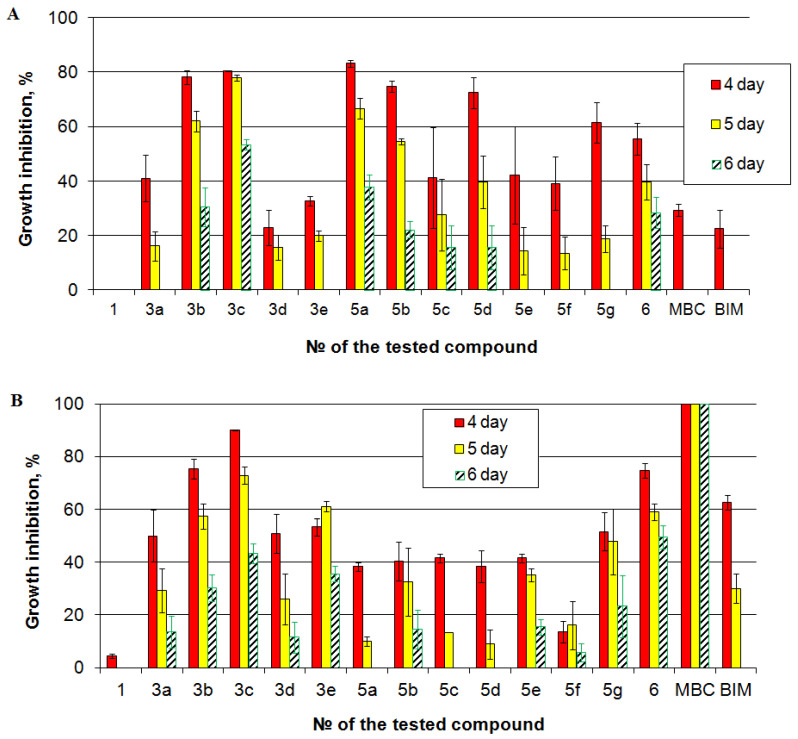
Fungistatic activity of the tested compounds determined on two isolates of *Microdochium nivale*: (**A**) isolate 1; (**B**) isolate 21. The presented values are averages ± standard error of at least three biological replicates. Red columns—4 day of incubation, yellow columns—5 day of incubation, hatched columns—6 day of incubation.

In our study, two genetically distinct isolates of *M. nivale* from the culture collection of FRC Kazan Scientific Center of RAS were used to test fungistatic activity of benzofuroxans. The parent compound (4,6-dichloro-5-nitrobenzofuroxan) was not effective against the analyzed isolates of *M. nivale*: it caused the reduction in fungal growth only during first three days of incubation. All of the tested benzofuroxan derivatives were more efficient than the parental compound (Figure 5). The most effective ones were the derivatives **3b**, **3c**. They caused more than 40% and 25% of growth inhibition on the fifth and sixth day of incubation, respectively, on both isolates. The least effective one was the derivative **5f**: it caused less than 20% and 10% of growth inhibition on the fifth and sixth day of incubation, respectively, on both isolates. Interestingly, two derivatives displayed isolate-specific fungistatic activity. The derivative **5a** was one of the most effective against isolate 1, but on isolate 21, it caused only 10% of inhibition on the fifth day of incubation and did not cause any inhibition by the sixth day of incubation. Contrarily, the derivative **3e** caused one of the most pronounced inhibitory effects on isolate 21 but was inefficient against isolate 1.

To compare the effects of benzofuroxans with that of the traditionally applied fungicides, benzimidazole (**BIM**) and carbendazim (methyl-2-benzimidazole carbamate, **MBC**) were used. Both carbendazim and benzimidazole had null inhibitory effect on isolate 1 by the fifth day of incubation (Figure 5). Herewith, on isolate 21, carbendazim caused 100% growth inhibitory effect till the sixth day of incubation. The effect of benzimidazole was lower than that of benzofuroxan derivatives **3b**, **3c**. Thus, although carbendazim is able to cause strong fungistatic effect against one isolates, there are isolates that display pronounced resistance to this compound. This is consistent with widely shown carbendazim-resistance of plant pathogenic [54,55,56]. In turn, the effect of some benzofuroxan derivatives (**3b**, **3c**) is likely to be more universal against different varieties of *M. nivale*.

**Figure 6:**
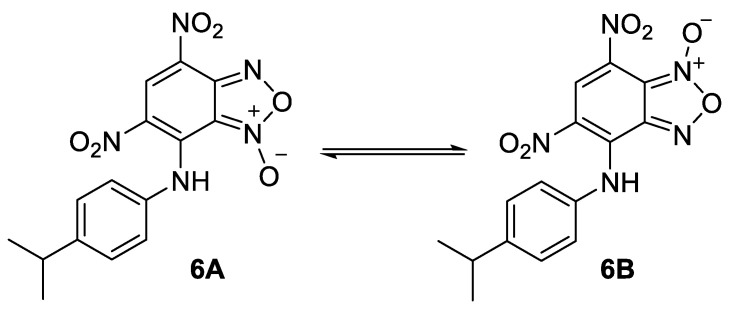
Structure of the tautomers of compound **6**.

For the comparison purposes we have also obtained the compound **6** as a mixture of tautomers (Figure 6) by the reaction of 7-chloro-4,6-dinitrobenzofuroxan with 4-isopropylaniline (see experimental part for the synthesis details). Its activity turned out to be comparable to that of lead compounds.

#### 2.2.3. Cytotoxicity of Test Compounds Toward Cancer and Normal Human Cell Lines

We have previously shown that compounds containing benzofuroxan fragments exhibit cytotoxic activity against some cancer cell lines [40]. In this regard, a series of new amino-containing benzofuroxan derivatives was tested for cytotoxicity against normal (Chang liver—normal human liver cells) and human cancer cell lines (M-HeLa—epithelioid carcinoma of the cervix; MCF-7—breast adenocarcinoma cells human; PANC-1—human pancreatic cancer cell line, glioblastoma cell line (T98G)).

The compounds tested showed high activity against cancer cell lines and low cytotoxicity against normal cells. The most significant results were obtained in the case of compounds **3c** and **3f** demonstrating selective cytotoxicity against the line of cervical carcinoma cells (M-HeLa) and breast adenocarcinoma cells human (MCF-7), which was comparable to the reference drug Doxorubicin and significantly exceeded Tamoxifen with an anti-cancer effect. Compounds **3d** and **3b** proved to be the most active against glioblastoma cell line (T98G). The IC_50_ values for these compounds were 14.7 and 12.7 μM, respectively. The selectivity of compounds for cancer cells is an important criterion for assessing the cytotoxic effect. For this, the selectivity index (SI) was calculated as the ratio between the IC_50_ value for normal cells and the IC_50_ value for cancer cells. The values IC_50_ and SI for the tested compounds are shown in Table 4.

The compounds with SI ≥ 3 are usually highly selective [57]. In this regard, it can be considered that compounds **3f** and **3c** are highly selective towards (MCF-7) and M-HeLa cell lines. The SI values for these lines were from 4.0 to 12.8. Doxorubicin and Tamoxifen reference drugs were far inferior to the leading compounds in selectivity.

The tested compounds were less active against human pancreatic cancer cell line (PANC-1). However, it is important to note that in relation to the line of normal liver cells, all tested compounds showed significantly lower toxicity compared to Doxorubicin and Tamoxifen.

#### 2.2.4. Induction of Apoptotic Effects by Test Compounds

Using the method of flow cytometry, studies were carried out that made it possible to establish whether the cytotoxic effect of the tested compounds is associated with the induction of apoptosis in cancer cells using the example of compounds of leaders **3c** and **3f.**

**Figure 7:**
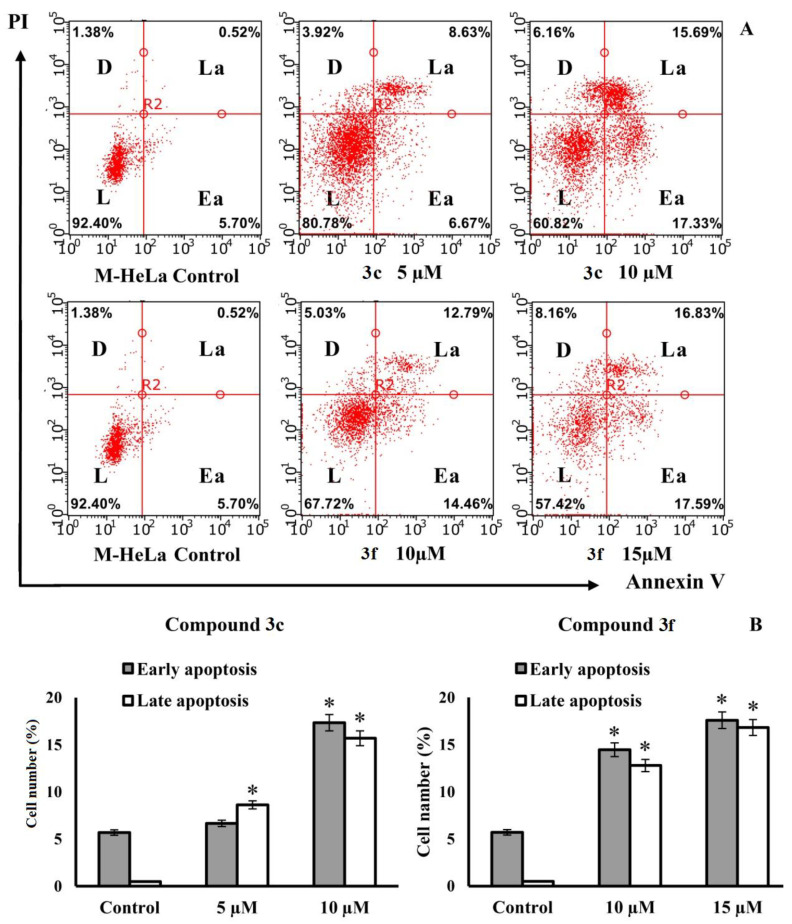
Apoptotic effects of compounds **3c** and **3f** on M-HeLa cells. (**A**) M-HeLa cells were treated with the indicated compounds at indicated concentrations for 24 h. Apoptotic effects were measured by flow cytometry using annexin V- Alexa Fluor 647 staining protocol. The values are presented as the mean ± SD (*n* = 3). L—living cells; D—dead cells; Ea—early apoptotic cells; La—late apoptotic cells. (**B**) Representative histograms for the numbers of cells (% of total) in the early and late stages of apoptosis for the control and experimental groups. The values are presented as the mean ± SD (*n* = 3): (*) *p* < 0.01 compared to control.

As shown in Figure 7, after 24 h of treatment, apoptotic effects were observed for both compounds in M-HeLa cells. At a concentration similar to the IC_50_ value, compound **3c** caused early and late apoptosis in 6.67% and 8.63% of M-HeLa cells, respectively, and compound **3f**, under the same conditions, caused early and late apoptosis in 14.46% and 12.79% of M-HeLa cells, respectively. It can be seen that compound **3f** at a concentration similar to the IC_50_ value induces apoptosis more actively than **3c**. The number of apoptotic cells at the stage of early apoptosis after treatment with **3f** increased by almost three times. When treated with compound **3c**, apoptosis in the early stage does not develop so actively (the number of apoptotic cells changes insignificantly compared to the control). With an increase in the concentration of compounds **3c** and **3f** to 10 μM and 15 μM, respectively, the number of cells in early and late apoptosis is approximately the same. The results suggest that the cytotoxic effects induced by compounds **3c** and **3f** in M-HeLa cancer cells are due to an apoptotic pathway.

#### 2.2.5. Effects on the Mitochondrial Membrane Potential (ΔΨ_m_)

Earlier, using Multiplex analysis and flow cytometry, we showed that compounds containing benzofuroxan fragments in the structure induce apoptosis, which most likely proceeds via an external pathway through the activation of death receptors located on the surface of cell membranes [40]. In this regard, studies were carried out to determine the external or internal mechanism of apoptosis caused by new derivatives of benzofuroxans in M-HeLa cells.

Studies were performed using flow cytometry methods using JC-10 reagent. In normal cells with a high potential of the mitochondrial membrane, the dye JC-10 forms aggregates (J-aggregate) near the mitochondrial membranes. When the membrane potential due to the stimulation of apoptosis falls, JC-10 is evenly distributed in the cell as a monomer (J-monomer). JC-10 units in normal cells have red fluorescence, and JC-10 monomers are green. The ratio between orange-red and green fluorescence can be used to assess the onset of apoptosis, which proceeds along the internal mitochondrial pathway. The experiments carried out confirmed the data obtained earlier.

**Figure 8:**
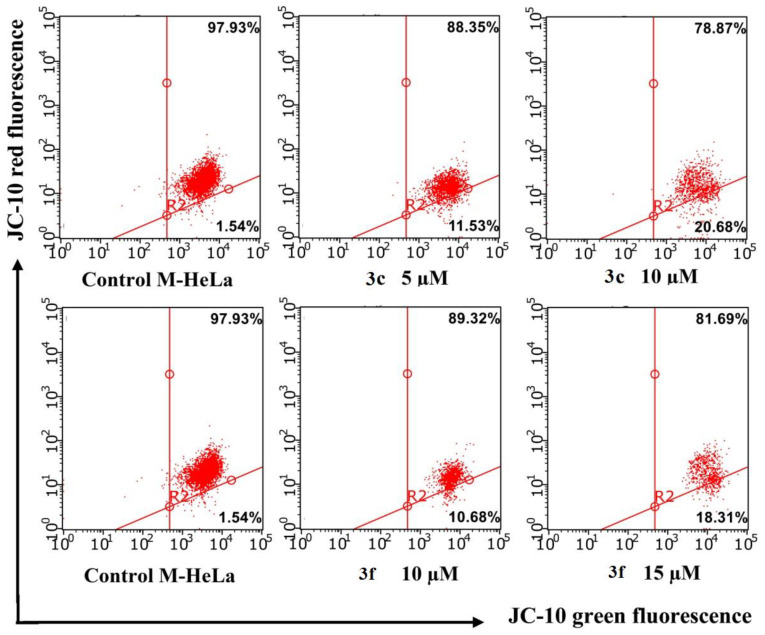
Flow cytometry analysis of M-Hela cells treated with compounds **3c** and **3f** along with the quantification of % of cells with red aggregates. The values are presented as mean ± SD.

A serious decrease in ΔΨ_m_ was not demonstrated by flow cytometry analysis (Figure 8). The intensity of red fluorescence after treatment of cells with compounds **5c** and **5g** at ≥ IC 50 concentrations did not change significantly compared to the control.

The results obtained confirm the data that the mechanism of action of benzofuroxan derivatives is not associated with the induction of apoptosis through the internal pathway.

#### 2.2.6. Cell Cycle Analysis 

Cell cycle analysis by quantification of DNA content is a reliable method to investigate at which phase cell cycle has been arrested, where in Propidium Iodide dye is used which binds in proportion to the amount of DNA present in the cell [58].

**Figure 9:**
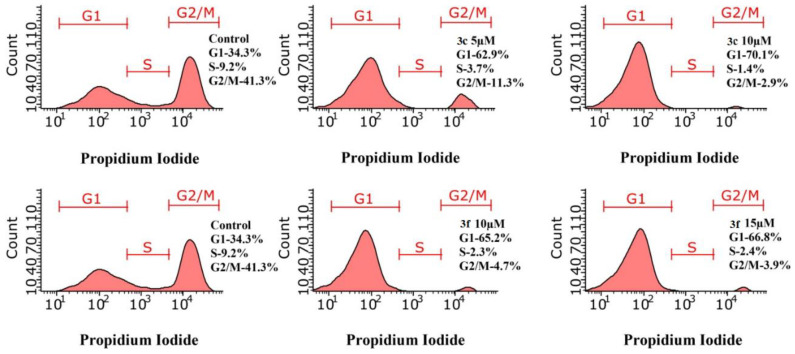
Effect of **3c** and **3f** on cell cycle progression of M-HeLa by flow cytometry.

The main checkpoint in the regulation of the cell cycle is the transition from the G1-phase to the S-phase. Depending on the amount of nutrients and energy, as well as on external factors, the cell decides whether to enter the cell cycle or go into a non-dividing state known as the G0 phase [59]. The results of cell cycle analysis using test compounds **3c** and **3f** against M-HeLa cell line by flow cytometry are shown in Figure 9. The data obtained demonstrate arrest of cells in the G0/G1 phase with a peak after 24 h. The tested compounds caused an increase in the% arrest in the G0/G1 phase. Compound **3c** by 62.91% and 70.06% at 5 and 10 μM and **3f** by 65. 2% and 66.8% at 10 and 15 μM, respectively, than control cells (34.28%).

## 3. Materials and Methods

### 3.1. Chemistry

IR spectra were recorded on a UR-20 spectrometer in the 400–3600 cm^−1^ range in KBr. ^1^H-NMR spectra were recorded on IR spectra were recorded on a UR-20 spectrometer in the 400–3600 cm^−1^ range in KBr. The ^1^H- and ^13^C-NMR spectra were recorded on a Bruker AVANCE 400 spectrometer (Bruker BioSpin, Rheinstetten, Germany) operating at 400 MHz (for ^1^H NMR) and 101 MHz (for ^13^C NMR), Bruker AVANCE 500 (Bruker BioSpin, Rheinstetten, Germany) spectrometer operating at 500 MHz (for ^1^H NMR) and 126 MHz (for ^13^C NMR) and Bruker Avance 600 spectrometer (Bruker BioSpin, Rheinstetten, Germany) operating at 600 MHz (for ^1^H NMR) and 151 MHz (for ^13^C NMR). Chemical shifts were measured in δ (ppm) with reference to the solvent (δ = 7.27 ppm and 77.00 ppm for CDCl_3_, δ = 2.06 ppm and 28.94 ppm for (CD_3_)_2_CO for ^1^H and ^13^C NMR, respectively). Elemental analysis was performed on a CHNS-O Elemental Analyser EuroEA3028-HT-OM (EuroVector S.p.A., Milan, Italy). The melting points were determined in glass capillaries on a Stuart SMP 10 instrument.

X-ray crystallography data: Single crystal X-ray diffraction data were collected on a Bruker Kappa Apex II CCD diffractometer using graphite monochromated MoK*α* (0.71073 Å) radiation and ω-scan rotation. Data collection: images were indexed, integrated, and scaled using the APEX2 [60] data reduction package and corrected for absorption using SADABS [61]. The structures **5a**-**5d** were solved by the direct methods and refined using SHELX [62]. Non-hydrogen atoms were refined anisotropically. Hydrogen atoms were calculated on idealized positions and refined as riding atoms. The X-ray analysis was performed on the equipment of Spectral-Analytical Center of FRC Kazan Scientific Center of RAS.

CCDC xxx (**5a**-**5d**) contain the supplementary crystallographic data for this paper. These data can be obtained free of charge via www.ccdc.cam.ac.uk/conts/retrieving.html (or from the Cambridge Crystallographic Data Centre, 12 Union Road, Cambridge CB2 1EZ, UK; fax: (+44) 1223-336-033; or deposit@ccdc.cam.uk).

Quantum-chemical computations. All quantum-chemical computations were carried out with the use of Gaussian 16 suite of programs [63]. Calculations were performed with Becke’s three parameter hybrid exchange functional [64] and the gradient-corrected nonlocal correlation functional of Lee et al. [65] (B3LYP) in combination with standard 6–31G* basis set [66,67,68]. For all compounds, geometry optimization of structures was performed without symmetry constraints. To ensure the calculated structures of reagents and products were indeed minima, vibrational analyses were performed using the same methods and were proved by all positive eigenvalues of Hessian matrix. The transition states were confirmed by the presence of one negative eigenvalue in the Hessian matrix of the second derivatives. All calculations were performed for a singlet surface and the solutions found were tested for stability against perturbations imposed on the wave function using the Stable procedure.

The following compounds were prepared following the literature procedures indicated: 4,6-dichloro-5-nitrobenzofuroxan **1** [69], 6-chloro-4-((3-hydroxyphenyl)amino)-5-nitrobenzofuroxan **3f** [40], 4-(butylamino)-6-chloro-5-nitrobenzofuroxan **5a**, 4-(benzyl(methyl)amino)-6-chloro-5-nitrobenzofuroxan **5f** and 6-chloro-5-nitro-4-(pyrrolidin-1-yl)benzofuroxan **5g** [70]. 

Reaction between 4,6-dichloro-5-nitrobenzofuroxan **1** and amines. To a solution of 4,6-dichloro-5-nitrobenzofuroxan **1** (0.0008 mol) in 5 mL of CHCl_3_ or DMSO at room temperature was added a solution of amine **3** (0.0016 mol) in 5 mL of CHCl_3_ or DMSO. The reaction was carried out at room temperature and under magnetic stirring, and the conversion was monitored through TLC analysis (eluent: toluene/ethyl acetate, 2:/1). After 2–4 h the crude mixture was precipitated in hexane (10 mL) from CHCl_3_ or water from DMSO, the obtained solid was filtered off, washed with cold water (100 mL) and dried under vacuum (0.06 mm Hg) at 40 °C temperature to constant weight. Crude product was purified by column chromatography (eluent: toluene/ethylacetate, 2/1) and recrystallized from chloroform/hexane (3/1) to give target compound. 

6-chloro-4-((4-fluorophenyl)amino)-5-nitrobenzofuroxan (**3a**). Purpule powder, yield 88%. M.p.: 166–168 °C. IR (*ν*, cm^−1^): 1206 (CF), 1368 (NO_2_ symm), 1558 (NO_2_ asymm), 1619 (furoxan ring), 3069 (C^7^H), 3441 (NH). ^1^H NMR (400 MHz, CDCl_3_) δ 8.60 (s, 1H), 7.25 (d, *J* = 4.5 Hz, 1H), 7.11 (m, *J* = 8.3 Hz, 3H), 6.89 (s, 1H).^13^C {1H} NMR (151 MHz, acetone-d_6_) δ 161.14 (d, *J* = 244.2 Hz), 147.47, 134.99, 132.53, 131.87, 127.33, 126.85 (d, *J* = 8.6 Hz), 115.50 (d, *J* = 23.1 Hz), 113.49, 101.91. Anal. calcd (%) for C_12_H_6_ClFN_4_O_4_: C 44.39; H 1.86; Cl 10.92; N 17.26. Found: C 44.41; H 1.78; Cl 10.88; N 17.34.

Crystal data: C_12_H_6_ClFN_4_O_4_ (*M* = 324.66 g/mol): monoclinic, space group *P*2_1_/*c* (no. 14), *a* = 9.2825(7), *b* = 19.3395(14), *c* = 7.2702(4) Å, *β* = 110.295(3)°, *V* = 1224.11(15)Å^3^, *Z* = 4, *T* = 173(2) K, F(000) 656, μ(MoKα) = 0.353 mm^−1^, *D_calc_* = 1.762 g/cm^3^, 10,745 reflections measured (2.1° ≤ θ ≤ 27.0°), 2670 independent (*R*_int_ = 0.043) which were used in all calculations, 199 parameters. The final *R*_1_ was 0.0382 (*I >* 2*σ*(*I*)) and *wR*_2_ was 0.1349 (all data), GOOF 0.94, largest diff. peak and hole 0.35 and −0.49 e.A^3^.

6-chloro-4-((4-chlorophenyl)amino)-5-nitrobenzofuroxan **(3b).** Red powder, yield 80%. M.p.: 134–135 °C. IR (*ν*, cm^−1^): 640 (CCl), 1360 (NO_2_ symm), 1559 (NO_2_ asymm), 1618 (furoxan ring), 3098 (C^7^H), 3444 (NH). ^1^H NMR (500 MHz, acetone-d_6_) δ 9.15 (s, 1H), 7.39 (m, 4H), 7.21 (s, 1H). ^13^C NMR (126 MHz, acetone-d_6_) δ 147.49, 137.93, 133.81, 130.94, 130.88, 128.80, 127.04, 125.46, 113.56, 102.88. Anal. calcd (%) for C_12_H_6_Cl_2_N_4_O_4_: C 42.25; H 1.77; Cl 20.79; N 16.43. Found: C 42.32; H 1.81; Cl 20.72; N 16.39.

Crystal data: C_12_H_6_Cl_2_N_4_O_4_ (*M* = 341.11 g/mol): monoclinic, space group *P*2_1_/*c* (no. 14), *a* = 9.4235(6), *b* = 19.9626(11), *c* = 7.2938(4) Å, *β* = 108.572(2)°, *V* = 1300.64(13) Å^3^, *Z* = 4, *T* = 173(2) K, F(000) 688, μ(MoKα) = 0.525 mm^−1^, *D_calc_* = 1.742 g/cm^3^, 11,863 reflections measured (2.0° ≤ θ ≤ 27.5°), 2987 independent (*R*_int_ = 0.042) which were used in all calculations, 199 parameters. The final *R*_1_ was 0.0388 (*I >* 2*σ*(*I*)) and *wR*_2_ was 0.1416 (all data), GOOF 0.95, largest diff. peak and hole 0.41 and −0.44 e.A^3^.

4-((4-bromophenyl)amino)-6-chloro-5-nitrobenzofuroxan (**3c**). Dark red powder, yield 77%. M.p.: 178–179 °C. IR (*ν*, cm^−1^): 683 (CBr), 1360 (NO_2_ symm), 1563 (NO_2_ asymm), 1621 (furoxan ring), 3094 (C^7^H), 3382 (NH). ^1^H NMR (400 MHz, acetone-d_6_) δ 9.14 (s, 1H), 7.53 (d, *J* = 8.6 Hz, 2H), 7.32 (d, *J* = 8.5 Hz, 2H), 7.22 (s, 1H). ^13^C {1H} NMR (101 MHz, acetone-d_6_) δ 147.48, 138.46, 134.03, 131.80, 130.69, 127.01, 125.64, 118.64, 113.58, 103.03. Anal. calcd (%) for C_12_H_6_BrClN_4_O_4_: C 37.38; H 1.57; Br 20.72; Cl 9.20; N 14.53. Found: C 37.45; H 1.64; Br 20.75; Cl 9.34; N 14.62.

Crystal data: C_12_H_6_BrClN_4_O_4_ (*M* = 385.56 g/mol): orthorhombic, space group *Pbca* (no. 61), *a* = 6.9497(5), *b* = 13.513(1), *c* = 28.0560(19) Å, *V* = 2634.8(3) Å^3^, *Z* = 8, *T* = 100(2) K, F(000) 1520, μ(MoKα) = 3.348 mm^−1^, *D_calc_* = 1.944 g/cm^3^, 18,129 reflections measured (3.3° ≤ θ ≤ 29.0°), 3507 independent (*R*_int_ = 0.052) which were used in all calculations, 199 parameters. The final *R*_1_ was 0.0297 (*I >* 2*σ*(*I*)) and *wR*_2_ was 0.1152 (all data), GOOF 0.83, largest diff. peak and hole 0.65 and −0.66 e.A^3^.

6-chloro-4-((4-cyanophenyl)amino)-5-nitrobenzofuroxan (**3d**). Compound **3d** was prepared as described above for other compounds using DMSO as solvent and heating 70 °C. The reaction mixture was stirred under heating for 2 h. Brown powder, yield 53%. M.p.: 160–161 °C. IR (*ν*, cm^−1^): 1360 (NO_2_ symm), 1554 (NO_2_ asymm), 1619 (furoxan ring), 2227 (CN), 3089 (C^7^H), 3390 (NH). ^1^H NMR (400 MHz, acetone-d_6_) δ 9.20 (s, 1H), 7.73 (d, *J* = 8.3 Hz, 2H), 7.51 (s, 1H), 7.46 (d, *J* = 7.9 Hz, 2H). ^13^C {1H} NMR (101 MHz, acetone-d_6_) δ 147.63, 144.33, 132.97, 128.27, 126.12, 121.57, 118.39, 113.88, 109.98, 107.19, 106.45. Anal. calcd (%) for C_13_H_6_ClN_5_O_4_: C 47.08; H 1.82; Cl 10.69; N 21.12. Found: C 47.15; H 1.78; Cl 10.59; N 21.16.

Crystal data: C_13_H_6_ClN_5_O_4_ (*M* = 331.68 g/mol): monoclinic, space group *P*2_1_/*c* (no. 14), *a* = 8.5796(7), *b* = 23.2320(17), *c* = 7.2254(6) Å, *β* = 111.777(4)°, *V* = 1337.40(19) Å^3^, *Z* = 4, *T* = 100(2) K, F(000) 672, μ(MoKα) = 0.317 mm^−1^, *D_calc_* = 1.647 g/cm^3^, 16,075 reflections measured (1.8° ≤ θ ≤ 27.0°), 2905 independent (*R*_int_ = 0.041) which were used in all calculations, 208 parameters. The final *R*_1_ was 0.0330 (*I >* 2*σ*(*I*)) and *wR*_2_ was 0.1228 (all data), GOOF 0.91, largest diff. peak and hole 0.37 and −0.30 e.A^3^.

6-chloro-4-((2-methoxyphenyl)amino)-5-nitrobenzofuroxan (**3e**) Red powder, yield 79%, m.p.: 133–134 °C. IR (*ν*, cm^−1^): 1308 (NO_2_ symm), 1562 (NO_2_ asymm), 1628 (furoxan ring), 3086 (C^7^H), 3331 (NH). ^1^H NMR (400 MHz, CDCl_3_) δ 8.61 (s, 1H), 7.33 (t, *J* = 7.9 Hz, 1H), 7.24 (d, *J* = 7.7 Hz, 1H), 6.98 (m, *J* = 7.7 Hz, 2H), 6.85 (s, 1H), 3.82 (s, 3H). ^13^C {1H} NMR (101 MHz, CDCl_3_) δ 153.39, 146.43, 133.98, 131.10, 128.75, 128.66, 126.36, 125.54, 120.60, 112.93, 111.46, 101.87, 55.74. Anal. calcd (%) for C_13_H_9_ClN_4_O_5_: C 46.38; H 2.69; Cl 10.53; N 16.64. Found: C 46.34; H 2.62; Cl 10.51; N 16.69.

6-chloro-4-(hexylamino)-5-nitrobenzofuroxan (**5b**). Dark red powder, yield 90%, m.p.: 95–96 °C. IR (*ν*, cm^−1^): 689 (CCl), 1332 (NO_2_ symm), 1535 (NO_2_ asymm), 1615 (furoxan ring), 3080 (C^7^H), 3430 (NH). ^1^H NMR (400 MHz, CDCl_3_), δ 8.36 (s, 1H), 6.61 (s, 1H), 4.06 (dd, *J* = 13.3, 7.0 Hz, 2H), 1.84–1.69 (m, 2H), 1.45 (dd, *J* = 14.5, 7.2 Hz, 2H), 1.36 (dt, *J* = 7.3, 4.8 Hz, 4H), 0.91 (dd, *J* = 9.7, 4.4 Hz, 3H). ^13^C {1H} NMR (101 MHz, CDCl_3_), δ 147.36, 138.39, 129.96, 126.98, 112.66, 99.41, 77.35, 77.03, 76.71, 47.45, 31.25, 30.13, 26.27, 22.43, 13.89. Anal. calcd (%) for C_12_H_15_ClN_4_O_4_: C 45.80; H 4.80; Cl 11.26; N 17.80. Found: C 45.84; H 4.82; Cl 11.25; N 17.76.

6-chloro-4-((3-morpholinopropyl)amino)-5-nitrobenzofuroxan (**5c**). Orange powder, yield 87%, m.p.: 110–111 °C. IR (*ν*, cm^−1^): 1335 (NO_2_ symm), 1536 (NO_2_ asymm), 1620 (furoxan ring), 3087 (C^7^H), 3435 (NH). ^1^H NMR (600 MHz, acetone-d_6_) δ 8.82 (s, 1H), 6.76 (s, 1H), 4.12 (m, 2H), 3.72–3.62 (m, 4H), 2.59–2.53 (m, 2H), 2.45 (m, 4H), 2.01–1.93 (m, 2H). ^13^C {1H} NMR (151 MHz, acetone-d_6_) δ 148.15, 136.88, 128.53, 127.90, 113.30, 98.14, 66.10, 57.38, 53.97, 46.67, 24.98. Anal. calcd (%) for C_13_H_16_ClN_5_O_5_: C 43.64; H 4.51; Cl 9.91; N 19.58. Found: C 43.56; H 4.54; Cl 9.93; N 20.01.

6-chloro-4-(cyclohexylamino)-5-nitrobenzofuroxan (**5d**). Orange powder, yield 94 %, m.p.: 113–115 °C. IR (*ν*, cm^–1^): 1330 (NO_2_ symm), 1530 (NO_2_ asymm), 1620 (furoxan ring), 3082 (C^7^H), 3436 (NH). ^1^H NMR (600 MHz, acetone-d_6_) δ 8.13–8.02 (s, 1H), 6.80 (s, 1H), 2.80–2.85 (m, 1H), 2.16 (d, *J* = 9.3 Hz, 2H), 1.83–1.77 (m, 2H), 1.59 (dd, *J* = 21.7, 9.6 Hz, 2H), 1.46 (dd, *J* = 25.2, 12.1 Hz, 2H). ^13^C {1H} NMR (151 MHz, acetone-d_6_) δ 147.71, 136.75, 128.70, 127.61, 113.38, 99.09, 55.43, 33.25, 24.96, 24.46. Anal. calcd (%) for C_12_H_13_ClN_4_O_4_: C 46.09; H 4.19; Cl 11.34; N 17.92. Found: C 46.05; H 4.23; Cl 11.29; N 17.96.

6-chloro-4-(morpholinoamino)-5-nitrobenzofuroxan (**5e**). Purpule powder, yield 42%, m.p.: 155–156 °C. IR (*ν*, cm^−1^): 1339 (NO_2_ symm), 1536 (NO_2_ asymm), 1624 (furoxan ring), 3077 (C^7^H), 3425 (NH). ^1^H NMR (600 MHz, CDCl_3_) δ 6.98 (s, 1H), 3.90–3.86 (m, 4H), 3.62–3.58 (m, 4H). ^13^C NMR {1H} (151 MHz, CDCl_3_) δ 148.91, 134.86, 127.78, 113.44, 106.82, 103.38, 66.65, 50.22. Anal. calcd (%) for C_10_H_10_ClN_5_O_5_: C 38.05; H 3.19; Cl 11.23; N 22.19. Found: C 38.07; H 3.22; Cl 11.25; N 22.17.

The mixture of 7-((4-isopropylphenyl)amino)-4,6-dinitrobenzofuroxan (6A) and 6-((4-isopropylphenyl)amino)-5,7-dinitrobenzofuroxan (6B).

To a solution of benzofuroxan (0.0008 mol) in chloroform (5 mL) at room temperature was added amine (0.0016 mol). The reaction mixture was stirred for 2 h. The solvent was evaporated *in vacuo* to give the crude product. Recrystallization from system chloroform/hexane (3:1) gave pure compound 7-(4-isopropylphenylamino)-4,6-dinitrobenzofuroxan **6**. Purpule powder, yield 42%, m.p.: 110–111 °C. IR (*ν*, cm^−1^): 1336 (NO_2_ symm), 1558 (NO_2_ asymm), 1625 (furoxan ring), 3385 (NH). Exists in solution as two tautomers. Major tautomer: ^1^H NMR (600 MHz, Acetone) δ 11.24 (s, 1H), 9.13 (s, 1H), 7.35 (dd, *J* = 23.0, 8.5 Hz, 4H), 3.00–295 (m, 1H), 1.27–1.26 (d, *J* = 6.9 Hz, 6H). ^13^C NMR (151 MHz, acetone-d_6_) δ 148.58, 146.89, 138.52, 136.94, 131.88, 127.32, 127.06, 126.06, 122.04, 111.44, 33.60, 23.23. Minor tautomer: ^1^H NMR (600 MHz, Acetone) δ 11.74 (s, 1H), 8.89 (s, 1H), 7.49 (d, *J* = 8.2, 4H), 2.78–2.80 (m, 1H), 1.30–1.29 (d, *J* = 7.0 Hz, 6H). ^13^C NMR (151 MHz, acetone-d_6_) δ 146.64, 148.15, 141.85, 135.73, 126.98, 126.95, 125.43, 124.63, 124.95, 106.46, 33.68, 23.33. Anal. calcd (%) for C_15_H_13_N_5_O_6_: C 50.14; H 3.65; N 19.49. Found: C 50.19; H 3.67; N 19.45.

### 3.2. Biological Studies

#### 3.2.1. Bacterial Biofilm Formation Inhibitory Activity

Materials and methods. Bacterial strain and cultivation conditions. For detection of biofilms formation strain *Vibrio aquamarinus* DSM 26054 was used. The strain forms biofilms, making this microorganism useful for studying biofilms.

*V. aquamarinus* DSM 26054 was cultivated for 24 h in LB medium [71], supplemented with 3% NaCl in the Innova 40R shaker incubator (New Brunswick Scientific, Enfield, CT, USA) at 25 °C and 200 rpm.

Chemicals. All of the chemicals used were of analytical grade. Crystal violet was obtained from Sigma-Aldrich (St. Louis, MO, USA). Azithromycin was obtained from Farmstandart (Moscow, Russia). Test solutions were prepared in deionized water immediately before the tests. 

The test compounds were dissolved in DMSO to the concentration of 1 × 10^−2^ M. Then they were diluted with ethanol. The control solutions were analogous dilutions of DMSO in ethanol. The tested compounds were also compared with the standard antibiotic Azithromycin. Azithromycin was dissolved in DMSO to the concentration of 5 × 10^−3^ M. Then it was diluted with deionized water. 

Test system for evaluation of biofilms production. To quantify the formation of biofilms the crystal violet assay with some modifications was used [72]. The necessary concentrations of the test compounds were prepared as described above.

Then, the suspensions of the daily culture of *V. aquamarinus* DSM 26054 was diluted with LB medium supplemented with 3% NaCl to the density of 10^8^ cells/mL.

The resulting suspension (190 μL) was added to the wells of a polystyrene microplate (Nuova Aptaca, Canelly, Regione Monforte, Italy). To some of the wells, 10 μL of the test substances at various concentrations were added. Since solvents used could also influence the biofilm formation, 10 μL of the appropriate solvent were added to the other part of the wells at same dilutions (control). Six replicates were done for each treatment and control. The microplate was covered with a lid and wrapped with a Parafilm (Bemis Company, Inc., Neenah, WI, USA).

After incubation at 25 °C for 72 h, biofilms were stained. The contents in the wells were removed by means of a dispenser. The wells were then carefully washed three times with 250 μL of sterile saline. The microplates were shaken to remove all non-adherent bacteria. Biofilms were fixed with 200 μL of 96% ethanol for 15 min. After the microplates had dried in air, 200 μL of 0.5% crystal violet were introduced into the wells. After 10 min, the dye was removed. The excess dye was removed by washing with water three times. After the microplates were air-dried, the dye in the wells bound to biofilms was dissolved with 200 μL of 96% ethanol. The extraction level (absorption) of crystal violet by ethanol was measured after 60 min at a wavelength of 570 nm using a FLUOstar Omega microplate reader (BMG Labtech, Ortenberg, Germany) in optical density units (OD570). The intensity of biofilm formation directly corresponds to the intensity of staining of the contents of the wells with the dye. Biofilm formation was determined by the difference between the mean OD readings obtained in the presence of compounds and the control.

Each experiment was performed in triplicate. The values were expressed as mean + SD. Student’s T-test was used to compare these values. Differences were considered statistically significant at *p* < 0.05.

#### 3.2.2. Fungistatic Activity of Benzofuroxans

Fungistatic activity of the tested compounds was assessed in vitro using agar growth medium poison technique [73,74,75]. The target compounds as well as widely used fungicides benzimidazole and carbendazim [54,55,56] were dissolved to 10 mM concentration in 20% Tween 20. The obtained solutions were added 1:9 *v*/*v* (giving 1 mM-concentrations of the target compounds) to potato-sucrose agar (PSA) [76] at 60 °C, mixed thoroughly and transferred to Petri dishes. For the control variants, PSA was supplemented with 20% Tween 20 (9:1). Fungistatic effect was tested against two isolates of *Microdochium nivale* causing snow mold disease of winter cereals (rye) from the culture collection of FRC Kazan Scientific Center of RAS. According to DNA sequence of ITS2 region, the isolates were genetically distinct (differed in ITS2 region sequence), but both belonged to the same species *Microdochium nivale* (paper under review). The center of each solidified medium was inoculated upside-down with 5-mm diameter mycelial plug cut from the periphery of 10-day-old cultures. The tested dishes were incubated at 20 °C. The percentage growth inhibition was calculated using Abbott’s formula (1):

%Inhibition = [(A − B) ÷ A] × 100(1)

where ‘A’ is mycelia growth in the control conditions and ‘B’ is mycelia growth in the treatment conditions. The assay was performed at least in triplicate. Linear growth of each colony was determined during 6 days.

#### 3.2.3. Cytotoxicity Assay

Cytotoxic effects of the test compounds on human cancer and normal cells were estimated by means of the multifunctional Cytell Cell Imaging system (GE Health Care Life Science, Uppsala, Sweden) using the Cell Viability Bio App which precisely counts the number of cells and evaluates their viability from fluorescence intensity [40]. Two fluorescent dyes that selectively penetrate the cell membranes and fluoresce at different wavelengths were used in the experiments. DAPI is able to penetrate intact membranes of living cells and colors nuclei in blue and Propidium iodide dye penetrates only dead cells with damaged membranes, staining them in yellow. IC_50_ was calculated using an online tool: MLA “Quest Graph™ IC_50_ Calculator.” AAT Bioquest, Inc, 25 July 2019, https://www.aatbio.com/tools/ic50-calculator. DAPI and propidium iodide were purchased from Sigma. The M-Hela clone 11 human, epithelioid cervical carcinoma, strain of Hela, clone of M-Hela; human breast adenocarcinoma cells (MCF-7); PANC-1—human pancreatic cancer cell line, glioblastoma cell line (T98G) from the Type Culture Collection of the Institute of Cytology (Russian Academy of Sciences) and Chang liver cell line (Human liver cells) from N. F. Gamaleya Research Center of Epidemiology and Microbiology were used in the experiments. The cells were cultured in a standard Eagle’s nutrient medium manufactured at the Chumakov Institute of Poliomyelitis and Virus Encephalitis (PanEco company, Moscow, Russia) and supplemented with 10% fetal calf serum and 1% nonessential amino acids. The cells were plated into a 96-well plate (Eppendorf, Hamburg, Germany) at a concentration of 10^5^ cells/mL, 150 μL of medium per well, and cultured in a CO_2_ incubator at 37 °C. Twenty-four hours after seeding the cells into wells, the compound under study was added at a preset dilution, 150 μL to each well. The dilutions of the compounds were prepared immediately in nutrient media; 5% DMSO that does not induce inhibition of cells at this concentration was added for better solubility. The experiments were repeated three times. Intact cells cultured in parallel with experimental cells were used as a control.

#### 3.2.4. Flow Cytometry Assay

Cell Culture. M-HeLa cells at 10^6^ cells/ well in a final volume of 2 mL were seeded into 6-well plates. After 24 h of incubation, various concentrations of compounds **3c** and **3f** were added to wells. 

Cell Apoptosis Analysis. The cells were harvested at 2000 rpm for 5 min and, then, washed twice with ice-cold PBS, followed by resuspension in binding buffer 100 μL. Next, the samples were incubated with 0.35 μL of annexin V-Alexa Fluor 647 and 0.1 μL of propidium iodide for 40 min at room temperature in the dark. Finally, the cells were analyzed by flow cytometry (Guava easyCyte, MERCK, Kenilworth, NJ, USA). The experiments were repeated three times.

#### 3.2.5. Mitochondrial Membrane Potential

The cells were harvested at 2000 rpm for 5 min and, then, washed twice with ice-cold PBS, followed by resuspension in JC-10 (10 µg/mL) and incubation at 37 °C for 10 min. After the cells were rinsed three times and suspended in PBS, the JC-10 fluorescence was observed by flow cytometry.

#### 3.2.6. Cell Cycle Analysis

The DNA content and cell-cycle distribution after genistein treatment were estimated by flow cytometry. Cell seeding, drug treatment and ethanol fixation were similar to cell proliferation assay. After washing with PBS, genistein and daidzein-treated and -fixed cells were suspended in 150 μL of PBS, then 0.5 mL phosphate-citrate buffer (0.05 M, pH = 4.0) was added and the suspension was incubated at room temperature for 5 min to facilitate the extraction of low molecular weight DNA. Following centrifugation the cells were resuspended in 150 μL DNA staining solution (20 μg/mL propidium iodide, 200 μg/mL DNase (RNase-free), and 0.1% Triton X-100) and incubated in the CO_2_ incubator (37 °C for 30 min). Cell cycle distribution was determined by fluorescence-activated cell sorting analysis of propidium iodide-stained ethanol-fixed cells using a Guava EasyCyte (Guava easyCyte, MERCK, Kenilworth, NJ, USA) [77]. 

#### 3.2.7. Statistical Analysis

IC_50_ was calculated using an online tool: MLA “Quest Graph™ IC_50_ Calculator”. AAT Bioquest, Inc, 25 July 2019, https://www.aatbio.com/tools/ic50-calculator.

The cytometric results were analyzed by the Cytell Cell Imaging multifunctional system using the Cell Viability BioApp and Apoptosis BioApp application. The data in tables and graphs are given as the mean ± standard error.

## 4. Conclusions

Series of novel 4-aminobenzofuroxans was synthesized through nucleophilic aromatic substitution reaction of 4,6-dichloro-5-nitrobenzofuroxan. The regioselectivity of chlorine atom substitution only in the 4 position was first proved using the quantum chemistry calculations. The substitution of this chlorine atom is favored by the greater stability of the reaction product. Additionally, the formation of the 4-substituted product proceeds with the lower activation barrier. So, both thermodynamic and kinetic factors make this pathway preferable. The biological activity of the obtained compounds was also evaluated. Thus, some of the obtained compounds inhibit effectively bacterial biofilm growth. Moreover, the importance of the introduction of aromatic amines fragments was shown in comparison with derivatives containing fragments of aliphatic amines in the study of anti-fungal and anti-cancer activities. The effect of some benzofuroxan derivatives is likely to be more universal against different varieties of *M. nivale* fungi compared with benzimidazole and carbendazim. Additionally, 4-aminofuroxans possessing aniline moiety exhibit a high selectivity towards MCF-7 and M-HeLa tumor cell lines. At the same time, they are significantly less toxic towards normal liver cells compared to Doxorubicin and Tamoxifen. Thus, these compounds are promising candidates for further development both as anti-cancer and anti-microbial agents.

## Figures and Tables

**Table 1:** Relative energies (ΔE, kcal/mol), enthalpies (ΔH, kcal/mol) and Gibbs energies (ΔG, kcal/mol) of products **Pa** and **Pb** with HCl compared to initial reagents 4,6-dichloro-5-nitrobenzofuroxan **1** with aniline **A** (Scheme 3).

Reaction Products	ΔE	ΔH	ΔG
**Pa + HCl**	9.91	11.13	8.21
**Pb + HCl**	2.32	3.70	1.37

**Table 2:** Relative energies (ΔE, kcal/mol), enthalpies (ΔH, kcal/mol), Gibbs free energies (ΔG, kcal/mol) and Highest occupied molecular orbital–lowest unoccupied molecular orbital (HOMO-LUMO) gaps (eV) for products **Pa** and **Pb**, reagents, pre-reaction complexes and transition states of the reaction of 4,6-dichloro-5-nitrobenzofuroxan **1** with aniline **A**.

	ΔE	ΔH	ΔG	HOMO-LUMO
1 + A*	0.00	0.00	0.00	–
**R_(1 + A)_a**	−3.41	−2.09	5.69	–
**R_(1 + A)_b**	−3.20	−1.90	6.46	–
**WM1**	15.02	16.28	30.80	3.10
**WM2**	29.61	30.75	44.41	2.13
**Pa + HCl**	−9.91	−11.13	−8.21	2.99
**Pb + HCl**	−2.32	−3.70	−1.37	2.86

**Table 3:** Biofilm formation (%) by *V. aquamarinus* DSM 26054 in the presence of benzofuroxan derivatives in reference to control (control = 100%).

Compound	Biofilm Formation, %					
	Compound Concentration, M					
	1 × 10^−9^	1 × 10^−8^	1 × 10^−7^	1 × 10^−6^	1 × 10^−5^	1 × 10^−4^
**3a**	104.09	81.39	51.29 *	104.19 *	116.74	108.56 *
**3b**	29.56 *	18.75 *	49.17 *	98.92	46.33 *	5.78 *
**3c**	83.88	22.91 *	67.33 *	91.23	121.82 *	6.23 *
**3d**	15.18 *	11.69 *	57.03 *	100.31	63.01 *	118.72 *
**3e**	85.02	85.82	41.01 *	96.34	65.93 *	94.48 *
3f **	66.58	9.31 *	11.34 *	43.56 *	96.57	98.97
**5b**	88.50	25.54 *	37.51 *	88.43 *	92.32	100.88
**5e**	26.21 *	21.15 *	70.11 *	96.38	116.36	110.85 *
**5f**	25.67 *	26.88 *	63.58 *	96.08	96.24	103.47
**5g**	45.78 *	22.40 *	27.37 *	79.81 *	108.84	107.18
**Azithromycin**	103.42	102.43	99.39	103.80	81.50 *	103.42

* Differences compared to the control samples are statistically significant, *t* criterion, *p* < 0.05; the solutions of appropriate solvent in ethanol with the same concentration were used as control in experiments with benzofuroxans; six replicates were done for each treatment and control; ** Data for compound **3f** were first published in [40].

**Table 4:** In vitro cytotoxic effects (IC_50_ μM) and selectivity index values (SI) of Test compound ^a^.

Test Compound	Cancer Cell Lines								Normal Cell Lines
	M-HeLa		MCF_7_		PANC-1		T98G		Chang Liver
	IC_50_	SI	IC_50_	SI	IC_50_	SI	IC_50_	SI	
**3a**	45.9 ± 3.7	2.0	>100	n.s.	>100	n.s	28.3 ± 2.3	3.3	93.8 ± 8.3
**3b**	60.6 ± 4.7	1.0	69.2 ± 5.7	n.s.	82.7 ± 7.3	n.s	12.7 ± 1.1	5.0	62.7 ± 5.5
**3c**	4.8 ± 0.4	12.8	3.7 ± 0.2	16.6	59.2 ± 4.6	1.0	20.4 ± 1.7	3	61.3 ± 5.1
**3d**	16.6 ± 1.4	>6	67.9 ± 5.7	>1.5	61.3 ± 5.1	>1.6	14.7 ± 1.2	>6.8	>100
**3e**	62.7 ± 5.4	1.0	79.5 ± 6.4	n.s.	50.0 ± 4.1	1.3	21.5 ± 1.8	3.0	62.7 ± 5.6
**3f**	16.0 ± 1.3	4.0	8.1 ± 0.7	8.0	>100	n.s.	59.8 ± 4.8	1.1	64.4 ± 5.7
**5a**	56.5 ± 4.5	1.8	>100	n.s.	>100	n.s.	22.7 ± 1.9	4.4	100
**5b**	60.8 ± 4.8	>1.6	>100	n.s.	50.0 ± 3.9	>2	65.0 ± 5.8	>1.5	>100
**5c**	49.3 ± 3.9	>2	>100	n.s.	38 ± 2.9	>2.6	>100	n.s.	>100
**5d**	61.0 ± 5.2	1.1	79.1 ± 6.2	n.s.	>100	n.s.	31.6 ± 2.5	2.1	67.1 ± 5.7
**5e**	61.6 ± 5.2	1.1	>100	n.s.	>100	n.s.	57.1 ± 4.3	1.1	65.2 ± 5.8
**5f**	62.2 ± 5.6	>1.6	>100	n.s.	>100	n.s.	>100	n.s.	>100
**5g**	>100	n.s.	>100	n.s.	>100	n.s.	>100	n.s.	>100
**Doxorubicin**	3.0 ± 0.1	1.0	3.0 ± 0.2	1.0	3.3 ± 0.1	1.0	6.8 ± 0.5	n.s.	3.0 ± 0.2
**Tamoxifen**	28 ± 2.5	1.5	25 ± 2.2	1.7	–				42.1 ± 3.5

^a^ The experiments were repeated for three times. The results are expressed as the mean ± standard deviation (SD); n.s.—no selectivity.

## Supplementary Materials

The following are available online at https://www.mdpi.com/1422-0067/21/21/8292/s1, Table S1, Figures S1–S3—quantum chemical modeling, Figures S4–S14—Anti-biofilm activity data, Figures S15–S34—copies of NMR spectra of all synthesized compounds.
