# Supplementary material for: Design of Novel 4-Aminobenzofuroxans and Evaluation of Their Antimicrobial and Anticancer Activity
Source: Int J Mol Sci. 2020 Nov 5;21(21):8292. doi: 10.3390/ijms21218292 (PMC7663979; doi:10.3390/ijms21218292)
Supplement: Supplementary file 1 [file ijms-21-08292-s001.pdf]

SUPPLEMENTARY MATERIALS

Design of novel 4-aminobenzofuroxans and evaluation of their antimicrobial and anticancer activity.

Elena Chugunova [1,2,*], Almir Gazizov [1,2*], Marina Sazykina [3], Nurgali Akylbekov [4*], Anastasiya Gildebrant [3], Ivan Sazykin [3], Alexander Burilov [1,2], Nurbol Appazov [4,5], Shorena Karchava [3], Maria Klimova [3], Alexandra Voloshina [1], Anastasia Sapunova [1], Syumbelya Gumerova [1], Ayrat Khamatgalimov [1], Tatiana Gerasimova [1], Alexey Dobrynin [1], Olga Gogoleva [2] and Vladimir Gorshkov [2]

1. Arbuzov Institute of Organic and Physical Chemistry, FRC Kazan Scientific Center, Russian Academy of Sciences, Akad. Arbuzov st. 8, Kazan, Tatarstan 420088, Russia;
2. Laboratory of Plant Infectious Diseases, FRC Kazan Scientific Center of Russian Academy of Sciences, Lobachevskogo st. 2/31, Kazan, Tatarstan 420111 Russia.
3. Southern Federal University, Stachki Avenue, 194/2, Rostov-on-Don 344090, Russia,
4. Korkyt Ata Kyzylorda University, Aiteke bie str., 29A, Kyzylorda 120014, The Republic of Kazakhstan
5. I. Zhakaev Kazakh Scientific Reseach Institute of Rice Growing, Abay avenue, 25B, Kyzylorda 120008, The Republic of Kazakhstan

* Correspondence: agazizov@iopc.ru (A.G.); chugunova.e.a@gmail.com (E.C.); nurgali_089@mail.ru (N.A.); Tel.: +7-843-272-7324 (A.G.); +7-843-272-7324 (E.C.); +7-724-223-1041 (N.A.)

Contents

Quantum chemical calculations 1-2
Anti-biofilm activity studies 3-8
Copies of NMR spectra 9-18

Quantum chemical calculations details

Calculations show that at the first stage pre-reaction complexes **R_{1+A}a** and **R_{1+A}b** are formed (ΔE ~ 3 kcal/mol, see Table 2 in main text), with the flat benzene rings of aniline and benzofuroxan being located almost parallel to each other, and the NH_2-group of aniline being located opposite to corresponding chlorine atom in benzofuroxan (Fig. S1).

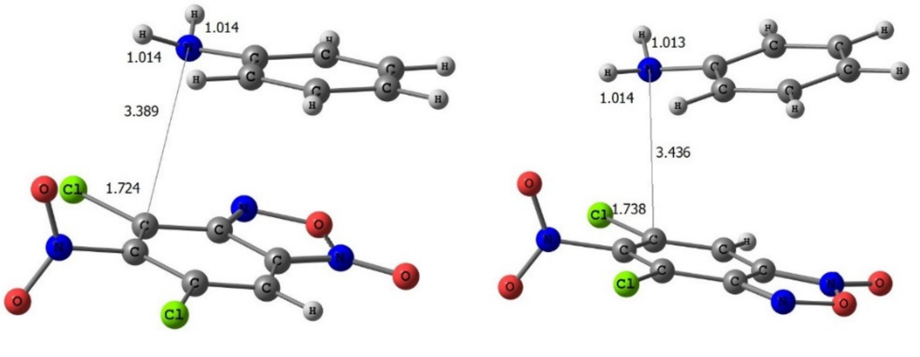

(a) *(b)*

Figure S1. The structure of the pre-reaction complexes **$R_{(1+A)}$a** (a) and **$R_{(1+A)}$b** (b) of the reaction of 4,6-dichloro-5-nitrobenzofuroxan **1** with aniline **A**

Further, pre-reaction complexes forms reaction products **Pa** and **Pb** through transition states **WM1** and **WM2** with activation energies of 15.02 and 29.61 kcal/mol, respectively (Fig. S2, zero is sum of the total energies of reactants **1** and **2** infinitely distant from each other), accompanied by the removal of hydrogen chloride.

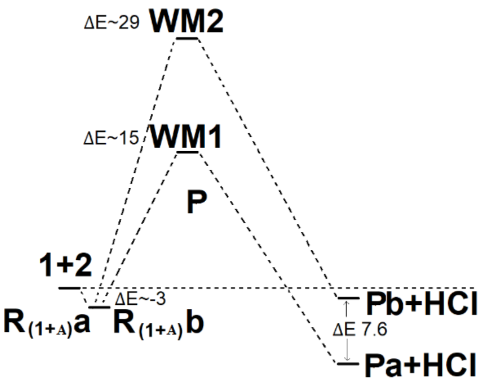

Figure S2. Energy diagram (ΔE, kcal/mol) of the reaction of 4,6-dichloro-5-nitrobenzofuroxan **1** with aniline **A**.

In the **WM1** and **WM2** transition states the distance between nitrogen atom of the aniline NH_2-group and the corresponding carbon atom of the benzofuroxan is shortened (1.8 Å compared to 3.4 in $\mathbf{R_{1+A}}$) and the C-Cl bond elongates (1.8-2.0 Å compared to 1.7 in $\mathbf{R_{1+A}}$), indicating subsequent remove of chlorine atom from benzofuroxan (Fig. S3, Table S1), leading to formation of products Pa and Pb (Fig. S4).

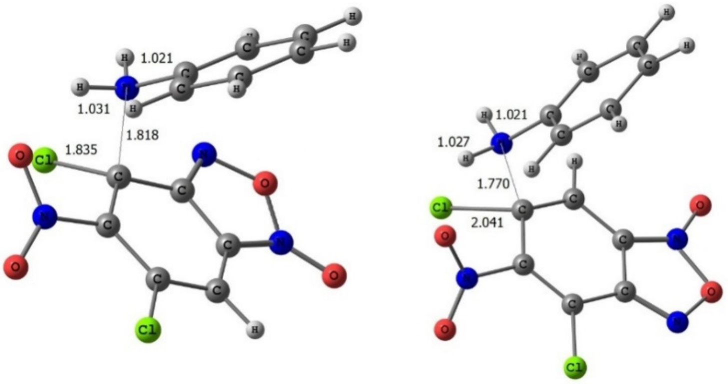

(a) *(b)*

Figure S3. The structure of the transition states **WM1** (a) and **WM2** (b) of the reaction of 4,6-dichloro-5-nitrobenzofuroxan **1** with aniline **A**.

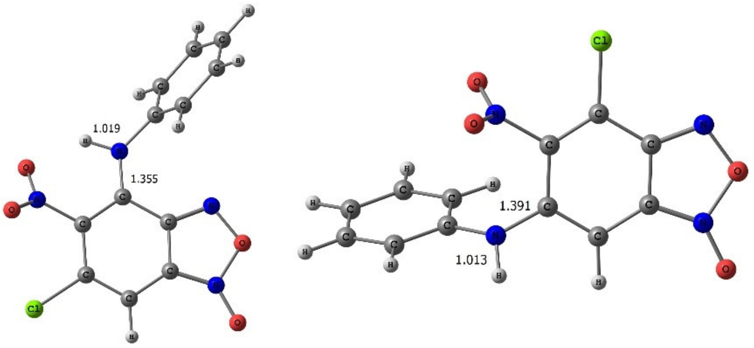

(**a**) (**b**)

Figure S4. The structure of **Pa** (a) and **Pb** (b).

Table S1. Structural parameters (bond lengths d and bond angles A) of the reactants and transition states of the reaction of 4,6-dichloro-5-nitrobenzofuroxan **1** with aniline **A**.

	**1***	**A**	$\mathbf{R_{(1+A)}a}$	$\mathbf{R_{(1+A)}b}$	**WM1**	**WM2**	**Pa**	**Pb**
$\mathbf{d_{C1\text{-}Cl}}$, **Å**	1.72	-	1.72	1.73	1.84	1.73	-	1.73
$\mathbf{d_{C4\text{-}Cl}}$, **Å**	1.74	-	1.74	1.74	1.75	2.04	1.74	-
$\mathbf{d_{C1\text{-}Nan}}$, **Å**	-	-	3.39	-	1.82	-	1.36	-
$\mathbf{d_{C4\text{-}Nan}}$, **Å**	-	-	-	3.44	-	1.77	-	1.39
$\mathbf{d_{Han\text{-}Cl}}$, **Å**	-	-	3.88, 3.95	3.91, 4.05	2.66, 2.72	2.52, 2.78	-	-
$\mathbf{d_{NH}}$, **Å**	-	1.01	1.01	1.01	1.02, 1.03	1.02, 1.03	1.02	1.01
$\mathbf{A_{CNH}}$, **°**	-	115	115	116	113	113	114	114

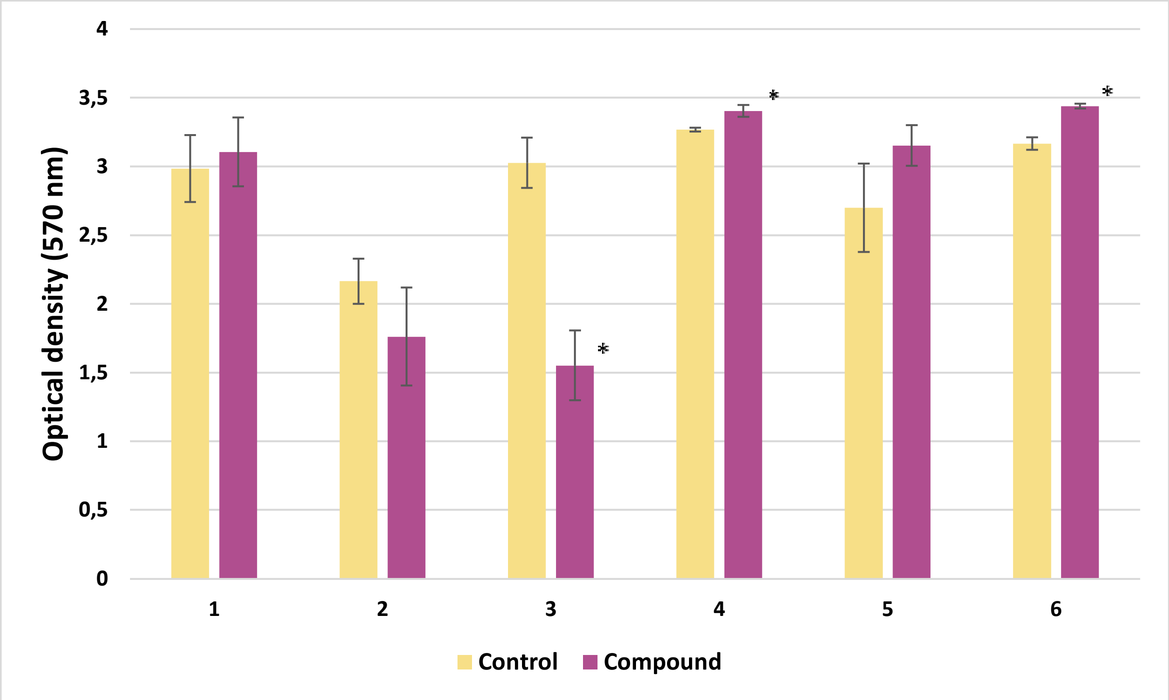

Figure S4. Antibiofilm activity of **3a** against *V. aquamarinus* DSM 26054: 1 – **3a** (1×10^{-9} M); 2 – **3a** (1×10^{-8} M); 3 – **3a** (1×10^{-7} M); 4 – **3a** (1×10^{-6} M); 5 – **3a** (1×10^{-5} M); 6 – **3a** (1×10^{-4} M). The solutions of appropriate solvent in ethanol with the same concentration were used as controls. Each experiment was performed in triplicate and repeated in six different occasions. The values were expressed as mean + SD. Student's T-test was used to compare these values. *Differences were considered statistically significant at $p < 0.05$.

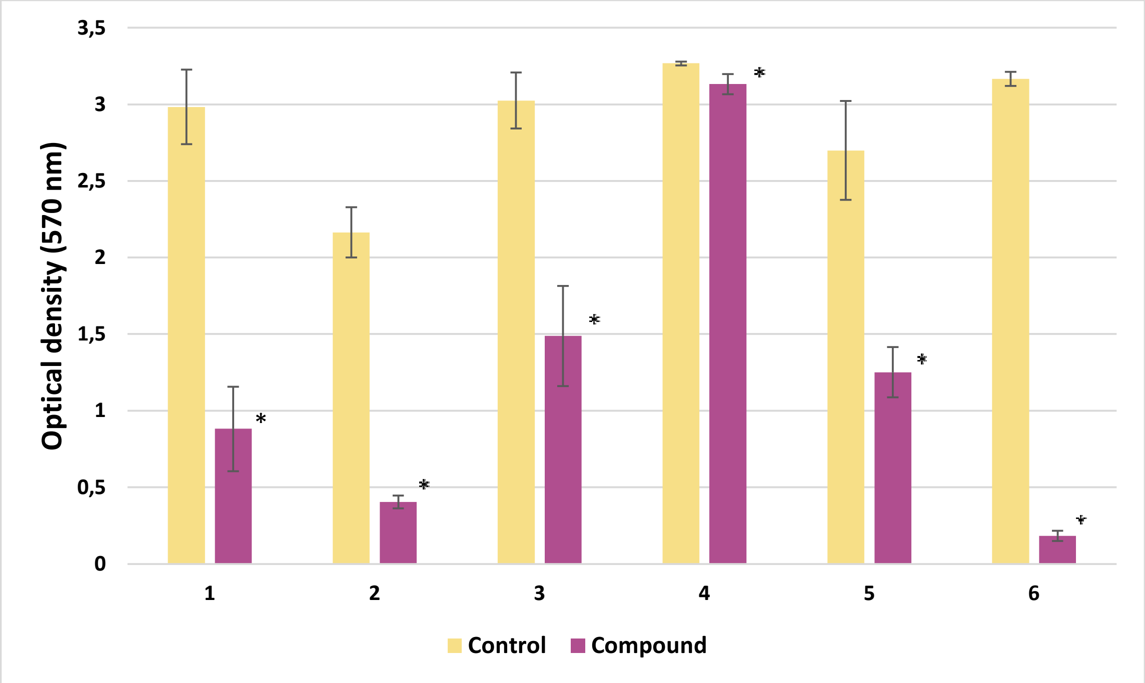

Figure S5. Antibiofilm activity of **3b** against *V. aquamarinus* DSM 26054: 1 – **3b** (1×10^{-9} M); 2 – **3b** (1×10^{-8} M); 3 – **3b** (1×10^{-7} M); 4 – **3b** (1×10^{-6} M); 5 – **3b** (1×10^{-5} M); 6 – **3b** (1×10^{-4} M). The solutions of appropriate solvent in ethanol with the same concentration were used as controls. Each experiment was performed in triplicate and repeated in six different occasions. The values were expressed as mean + SD. Student's T-test was used to compare these values. *Differences were considered statistically significant at $p < 0.05$.

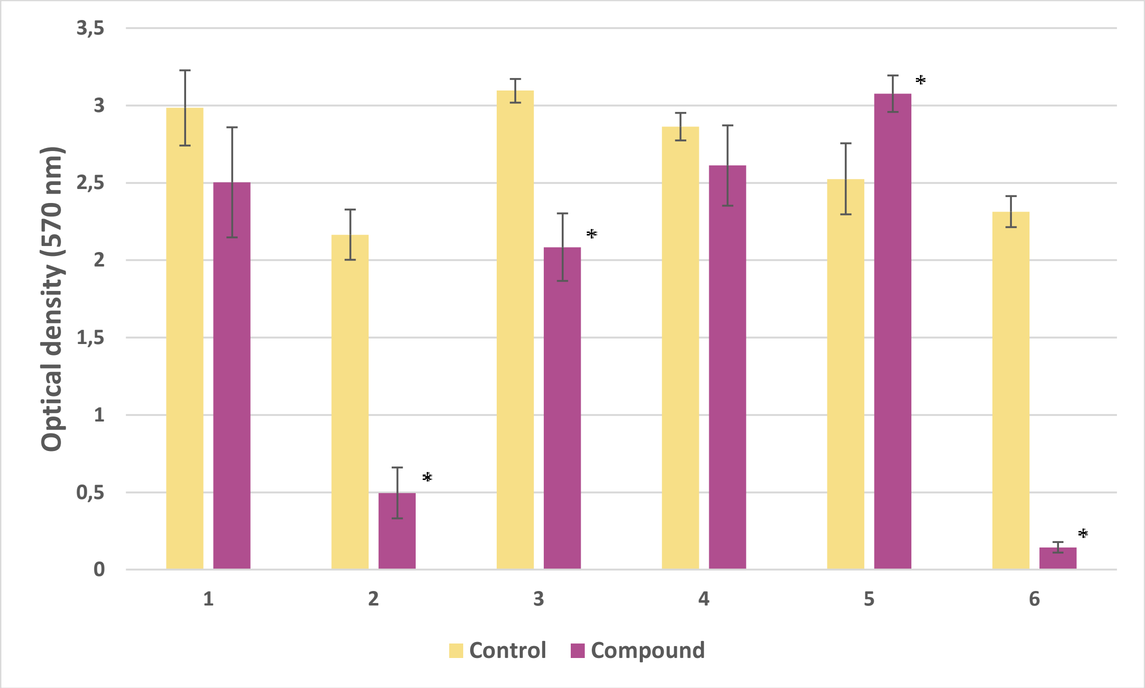

Figure S6. Antibiofilm activity of **3c** against *V. aquamarinus* DSM 26054: 1 – **3c** (1×10^{-9} M); 2 – **3c** (1×10^{-8} M); 3 – **3c** (1×10^{-7} M); 4 – **3c** (1×10^{-6} M); 5 – **3c** (1×10^{-5} M); 6 – **3c** (1×10^{-4} M). The solutions of appropriate solvent in ethanol with the same concentration were used as controls. Each experiment was performed in triplicate and repeated in six different occasions. The values were expressed as mean + SD. Student's T-test was used to compare these values. *Differences were considered statistically significant at $p < 0.05$.

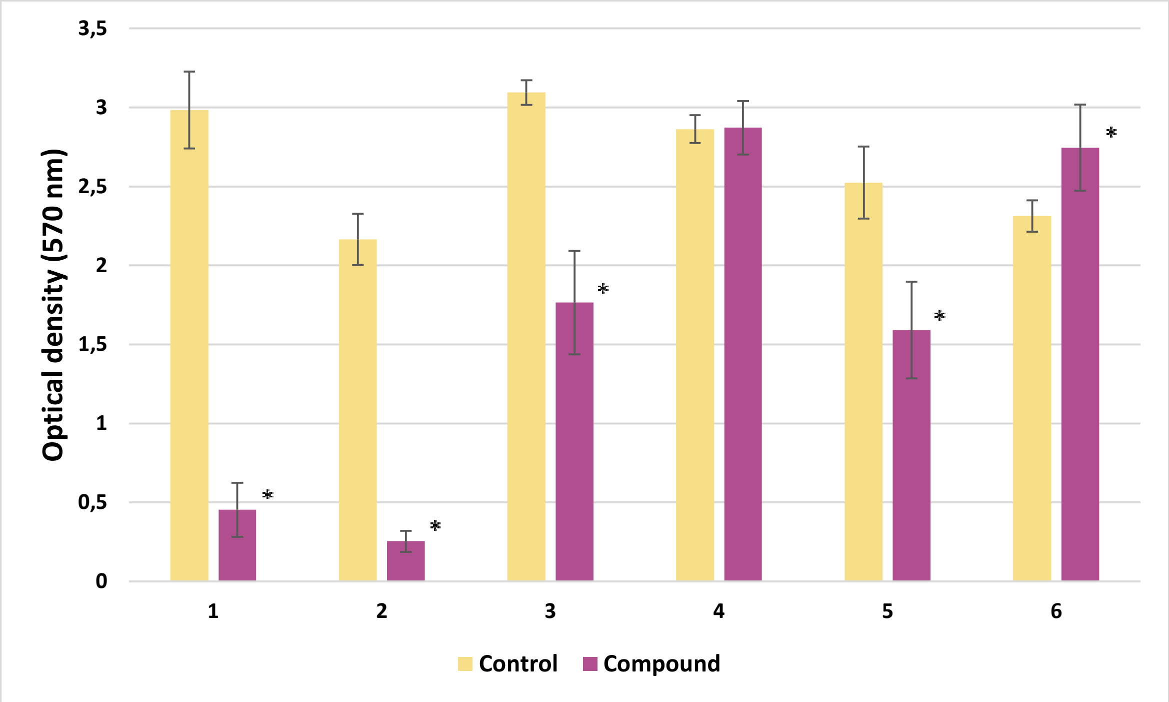

Figure S7. Antibiofilm activity of **3d** against *V. aquamarinus* DSM 26054: 1 – **3d** (1×10^{-9} M); 2 – **3d** (1×10^{-8} M); 3 – **3d** (1×10^{-7} M); 4 – **3d** (1×10^{-6} M); 5 – **3d** (1×10^{-5} M); 6 – **3d** (1×10^{-4} M). The solutions of appropriate solvent in ethanol with the same concentration were used as controls. Each experiment was performed in triplicate and repeated in six different occasions. The values were expressed as mean + SD. Student's T-test was used to compare these values. *Differences were considered statistically significant at $p < 0.05$.

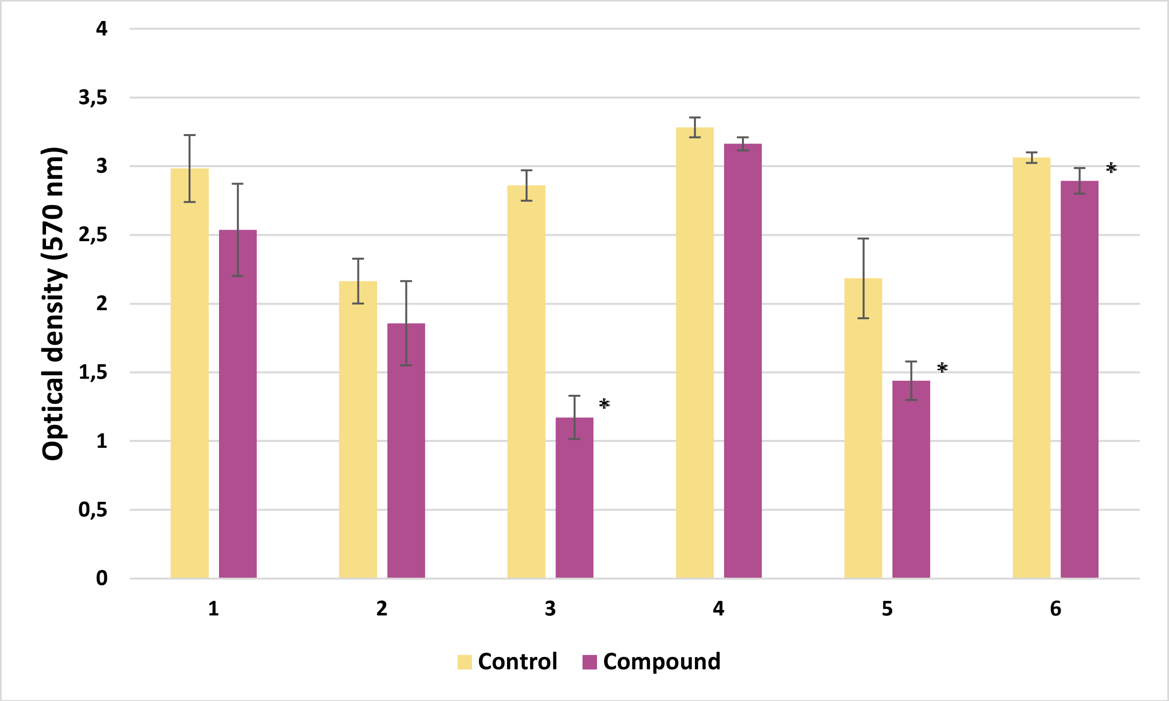

Figure S8. Antibiofilm activity of **3e** against *V. aquamarinus* DSM 26054: 1 – **3e** (1×10^{-9} M); 2 – **3e** (1×10^{-8} M); 3 – **3e** (1×10^{-7} M); 4 – **3e** (1×10^{-6} M); 5 – **3e** (1×10^{-5} M); 6 – **3e** (1×10^{-4} M). The solutions of appropriate solvent in ethanol with the same concentration were used as controls. Each experiment was performed in triplicate and repeated in six different occasions. The values were expressed as mean + SD. Student's T-test was used to compare these values. *Differences were considered statistically significant at $p < 0.05$.

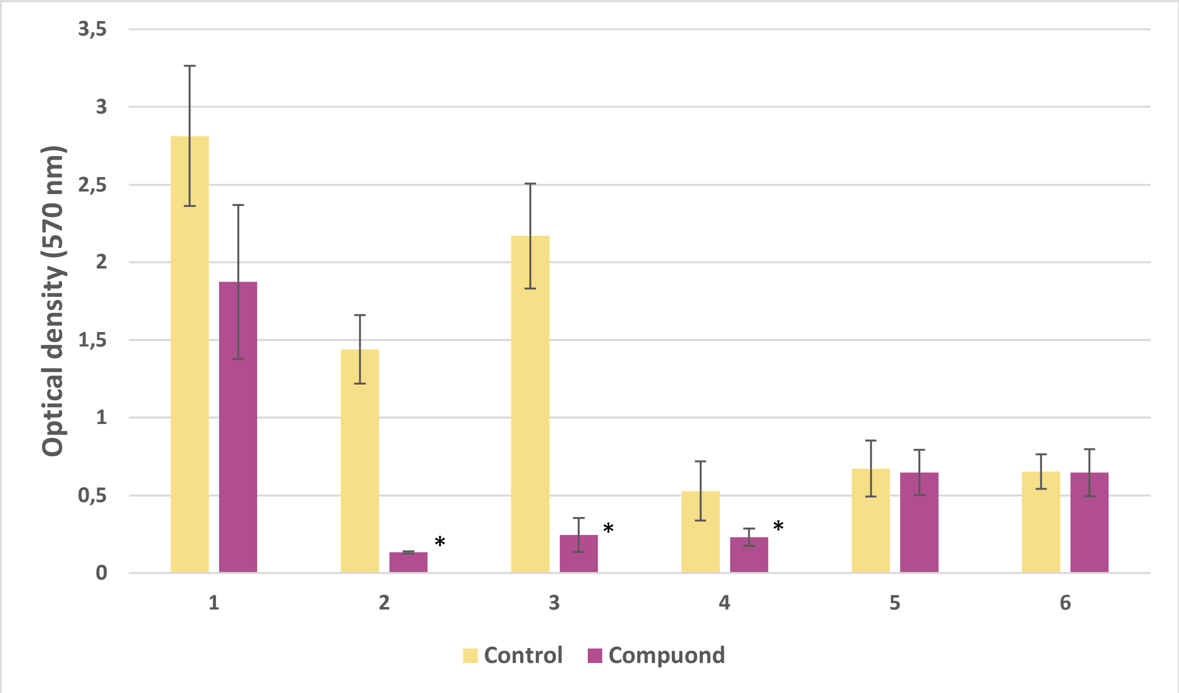

Figure S9. Antibiofilm activity of **3f** against *V. aquamarinus* DSM 26054: 1 – **3f** (1×10^{-9} M); 2 – **3f** (1×10^{-8} M); 3 – **3f** (1×10^{-7} M); 4 – **3f** (1×10^{-6} M); 5 – **3f** (1×10^{-5} M); 6 – **3f** (1×10^{-4} M). The solutions of appropriate solvent in ethanol with the same concentration were used as controls. Each experiment was performed in triplicate and repeated in six different occasions. The values were expressed as mean + SD. Student's T-test was used to compare these values. *Differences were considered statistically significant at $p < 0.05$.

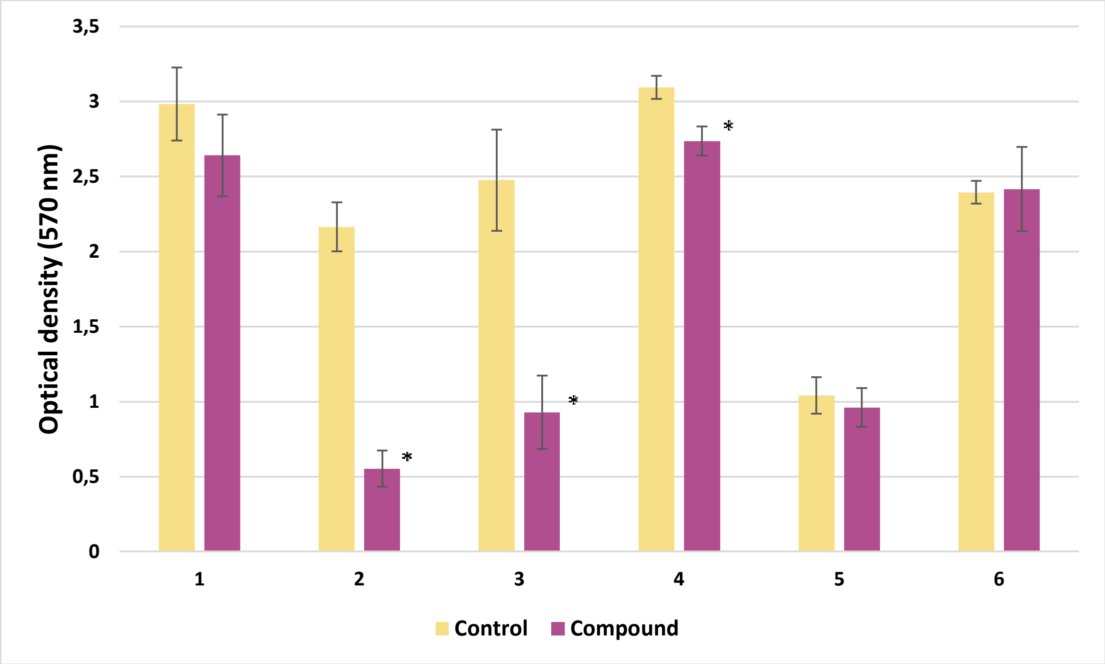

Figure S10. Antibiofilm activity of **5b** against *V. aquamarinus* DSM 26054: 1 – **5b** ($1{\times}10^{-9}$ M); 2 – **5b** ($1{\times}10^{-8}$ M); 3 – **5b** ($1{\times}10^{-7}$ M); 4 – **5b** ($1{\times}10^{-6}$ M); 5 – **5b** ($1{\times}10^{-5}$ M); 6 – **5b** ($1{\times}10^{-4}$ M). The solutions of appropriate solvent in ethanol with the same concentration were used as controls. Each experiment was performed in triplicate and repeated in six different occasions. The values were expressed as mean + SD. Student's T-test was used to compare these values. *Differences were considered statistically significant at $p < 0.05$.

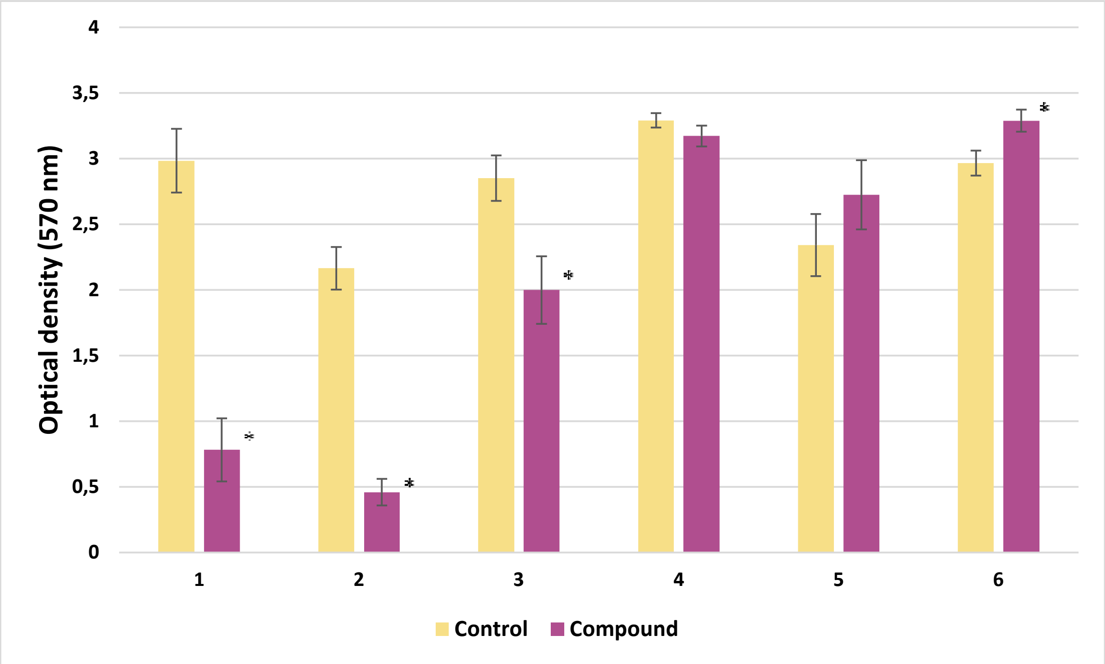

Figure S11. Antibiofilm activity of **5e** against *V. aquamarinus* DSM 26054: 1 – **5e** ($1{\times}10^{-9}$ M); 2 – **5e** ($1{\times}10^{-8}$ M); 3 – **5e** ($1{\times}10^{-7}$ M); 4 – **5e** ($1{\times}10^{-6}$ M); 5 – **5e** ($1{\times}10^{-5}$ M); 6 – **5e** ($1{\times}10^{-4}$ M). The solutions of appropriate solvent in ethanol with the same concentration were used as controls. Each experiment was performed in triplicate and repeated in six different occasions. The values were expressed as mean + SD. Student's T-test was used to compare these values. *Differences were considered statistically significant at $p < 0.05$.

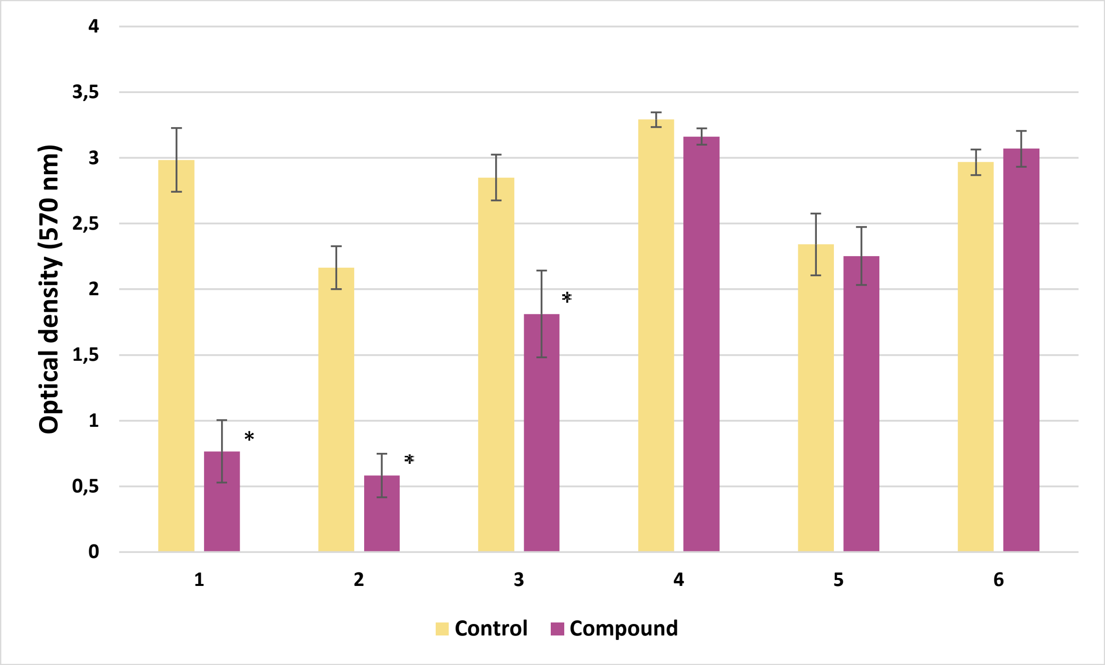

Figure S12. Antibiofilm activity of **5f** against *V. aquamarinus* DSM 26054: 1 – **5f** (1×10^{-9} M); 2 – **5f** (1×10^{-8} M); 3 – **5f** (1×10^{-7} M); 4 – **5f** (1×10^{-6} M); 5 – **5f** (1×10^{-5} M); 6 – **5f** (1×10^{-4} M). The solutions of appropriate solvent in ethanol with the same concentration were used as controls. Each experiment was performed in triplicate and repeated in six different occasions. The values were expressed as mean + SD. Student's T-test was used to compare these values. *Differences were considered statistically significant at $p < 0.05$.

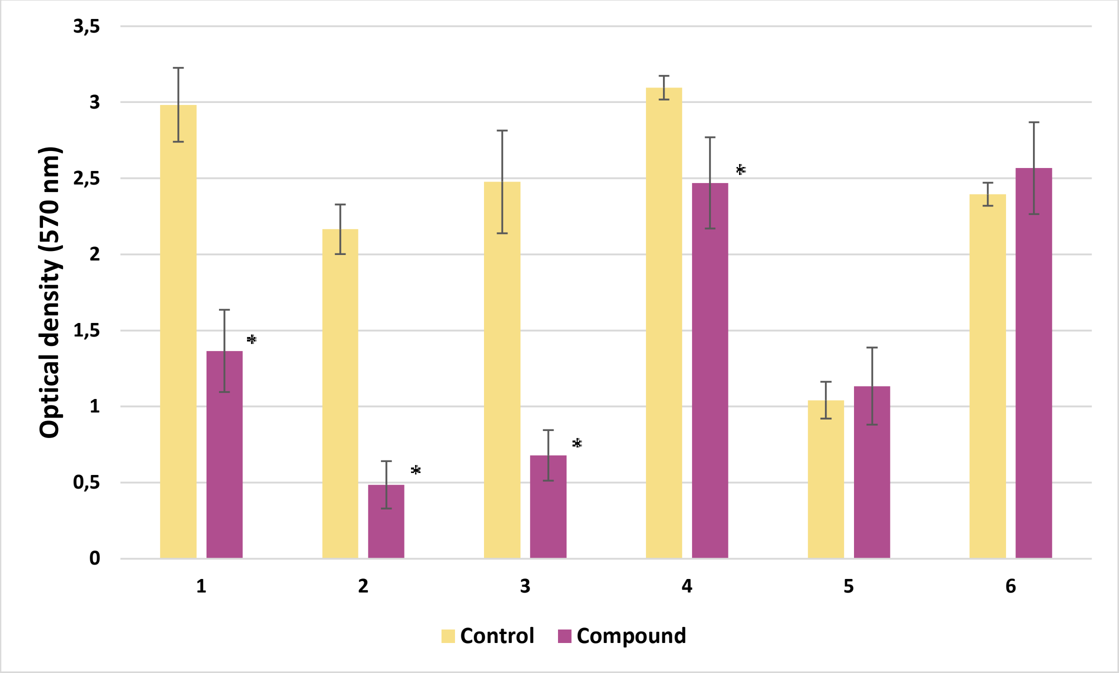

Figure S13. Antibiofilm activity of **5g** against *V. aquamarinus* DSM 26054: 1 – **5g** (1×10^{-9} M); 2 – **5g** (1×10^{-8} M); 3 – **5g** (1×10^{-7} M); 4 – **5g** (1×10^{-6} M); 5 – **5g** (1×10^{-5} M); 6 – **5g** (1×10^{-4} M). The solutions of appropriate solvent in ethanol with the same concentration were used as controls. Each experiment was performed in triplicate and repeated in six different occasions. The values were expressed as mean + SD. Student's T-test was used to compare these values. *Differences were considered statistically significant at $p < 0.05$.

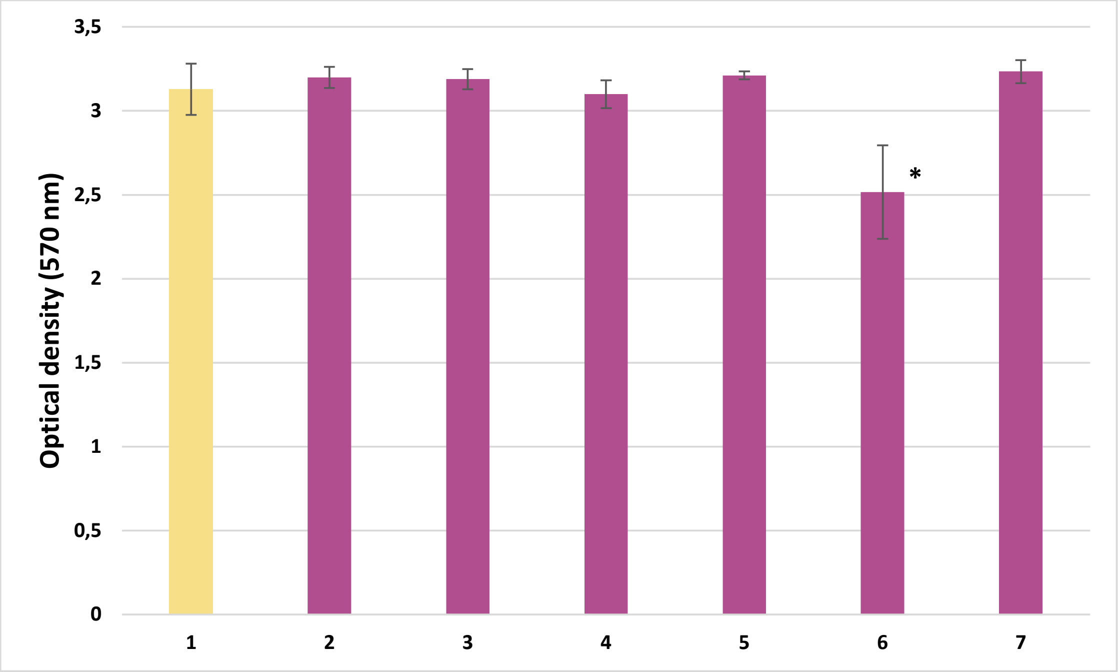

Figure S14. Antibiofilm activity of azithromycin against *V. aquamarinus* DSM 26054: 1 – control; 2 – azithromycin (1×10^{-9} M); 3 – azithromycin (1×10^{-8} M); 4 – azithromycin (1×10^{-7} M); 5 – azithromycin (1×10^{-6} M); 6 – azithromycin (1×10^{-5} M); 7 – azithromycin (1×10^{-4} M). Each experiment was performed in triplicate and repeated in six different occasions. The values were expressed as mean + SD. Student's T-test was used to compare these values. *Differences were considered statistically significant at $p < 0.05$.

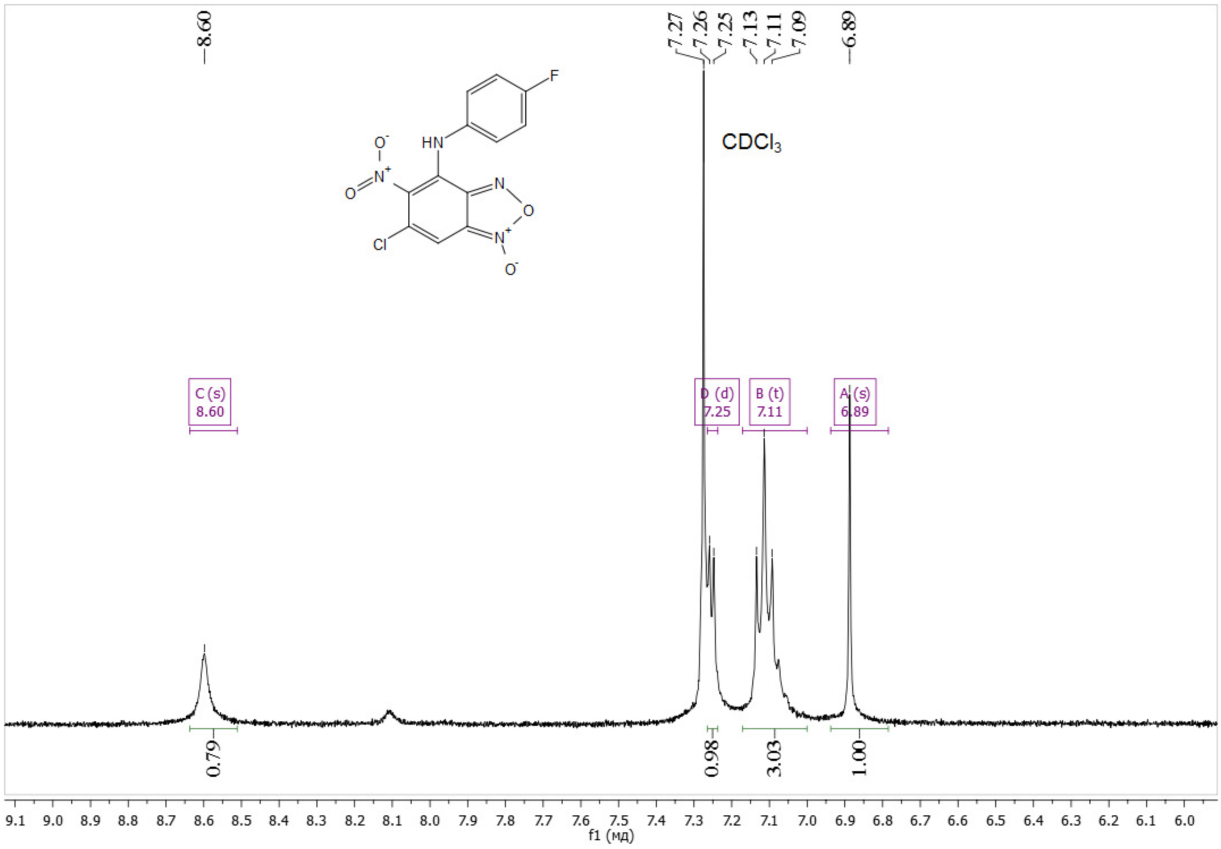

Figure S15. ^{1}H NMR ($CDCl_3$, 400 MHz, 25 °C) of compound **3a**.

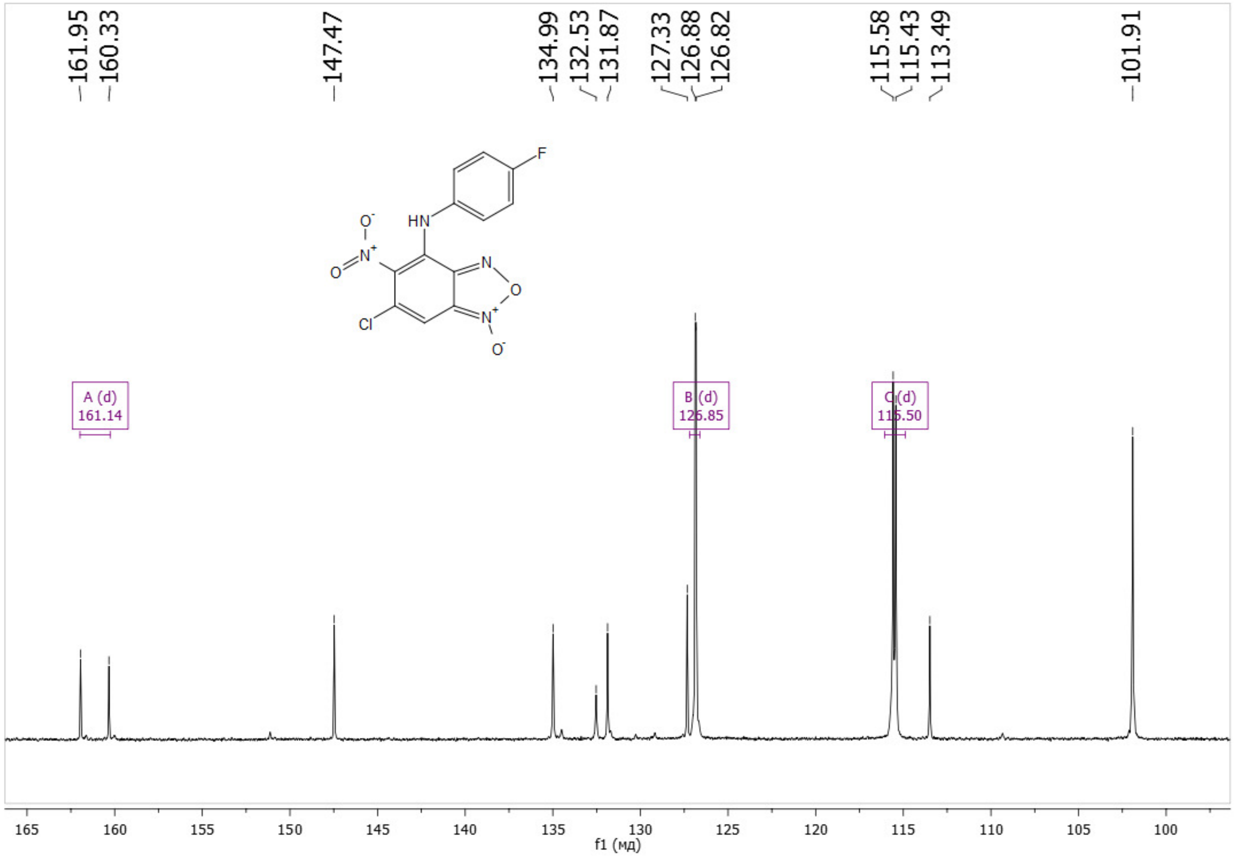

Figure S16. $^{13}C\{1H\}$ NMR (acetone-d_6, 151 MHz, 25 °C) of compound **3a**.

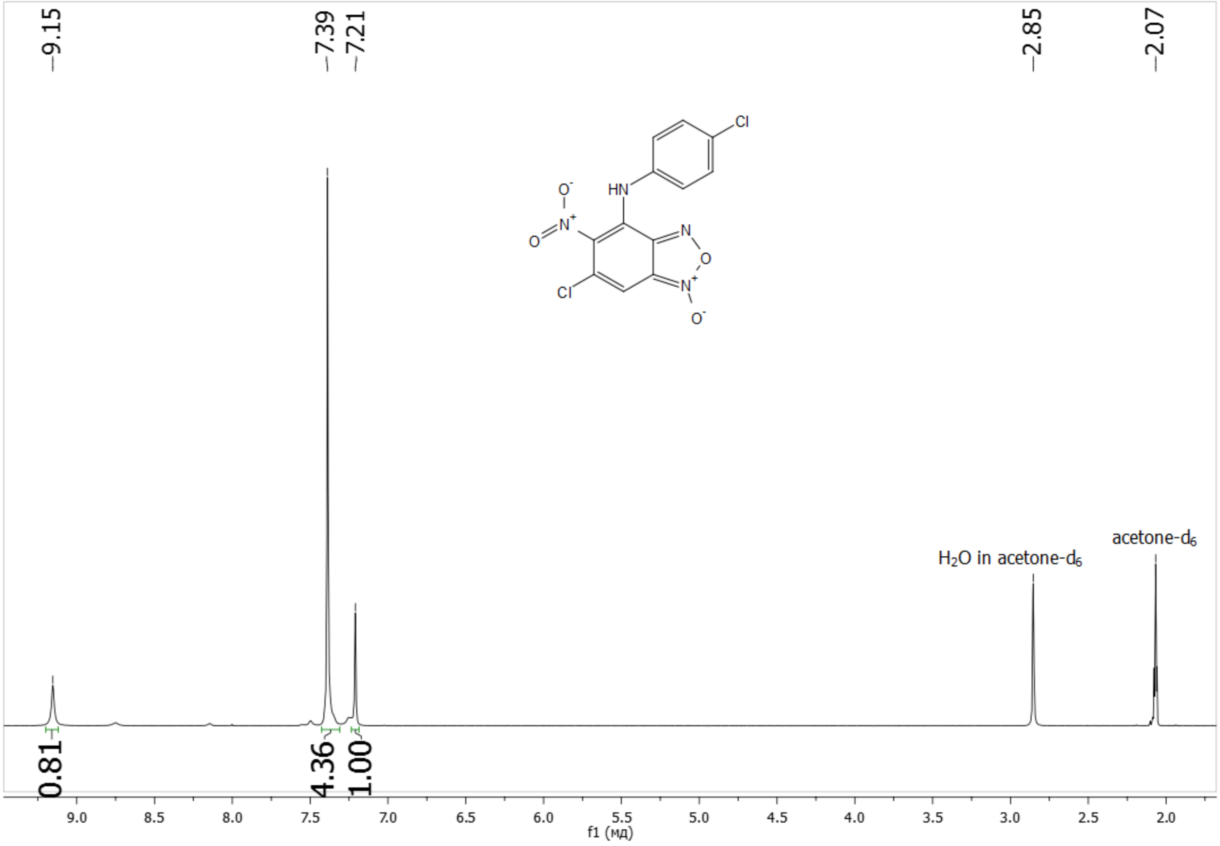

Figure S17. ^{1}H NMR (acetone-d_6, 500 MHz, 25 °C) of compound **3b**.

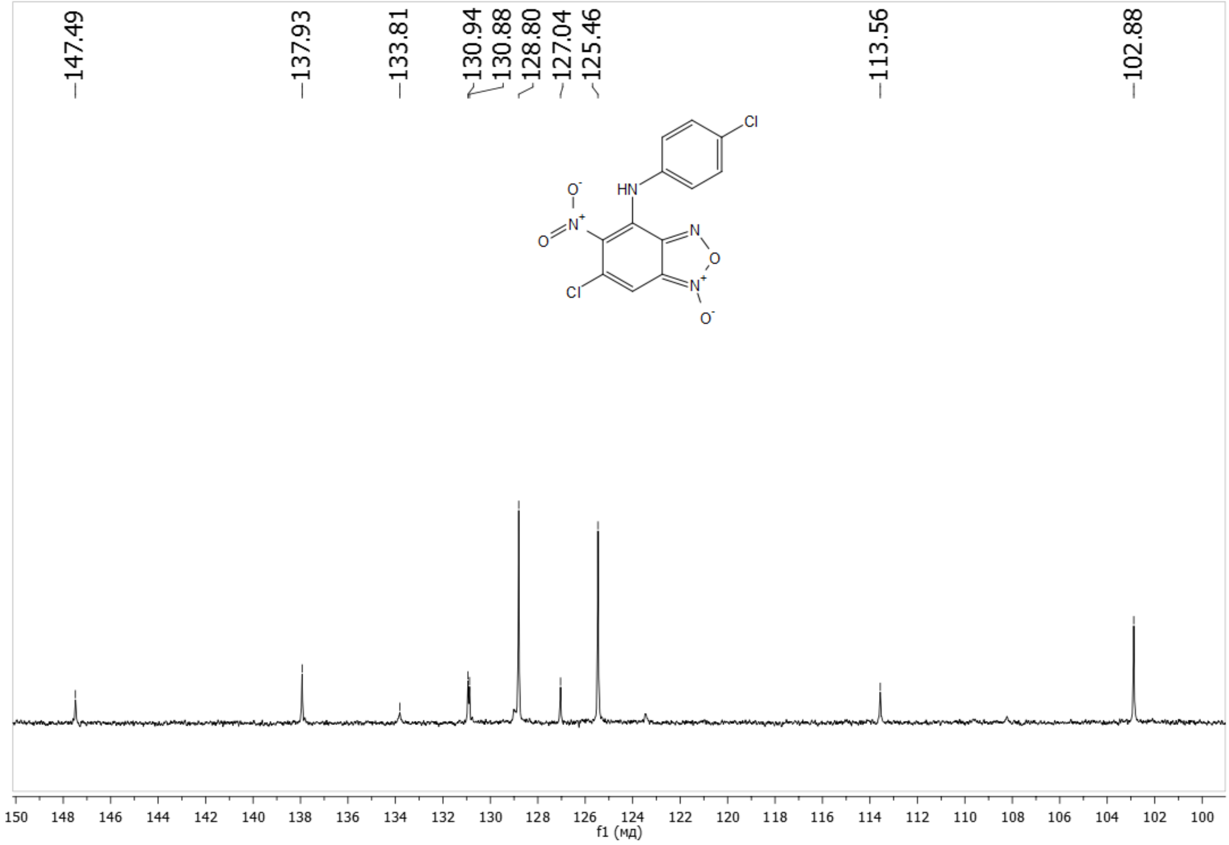

Figure S18. $^{13}C\{1H\}$ NMR (acetone-d_6, 126 MHz, 25 °C) of compound **3b**.

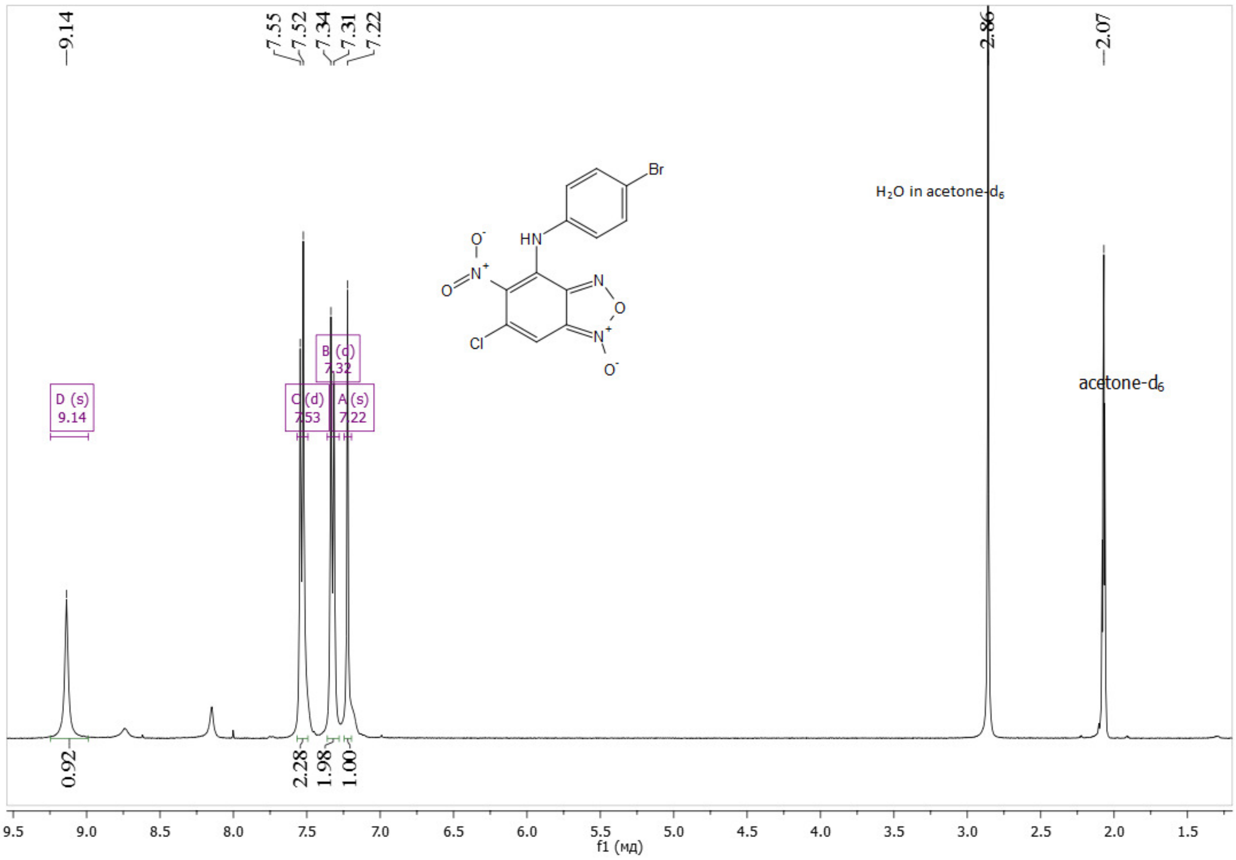

Figure S19. ^{1}H NMR (acetone-d_6, 400 MHz, 25 °C) of compound **3c**.

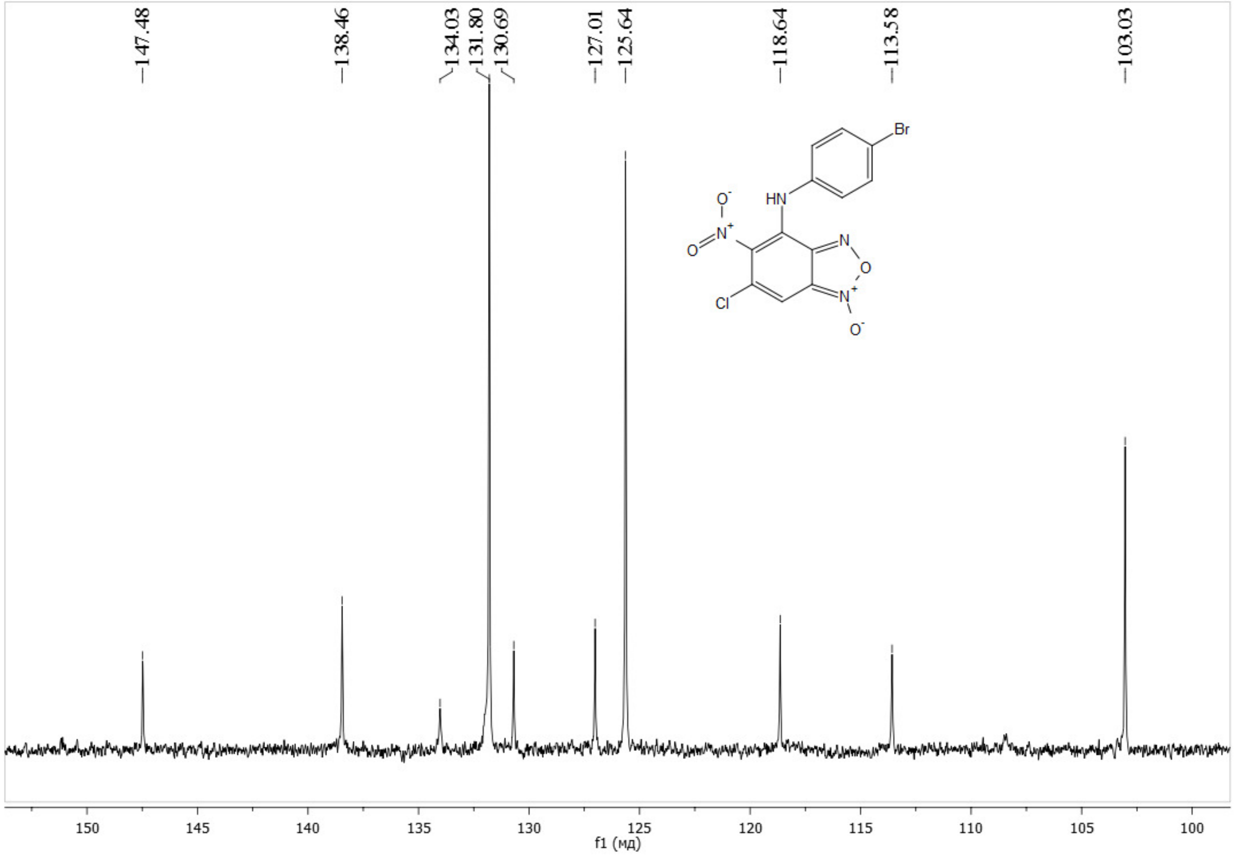

Figure S20. $^{13}C\{1H\}$ NMR (acetone-d_6, 151 MHz, 25 °C) of compound **3c**.

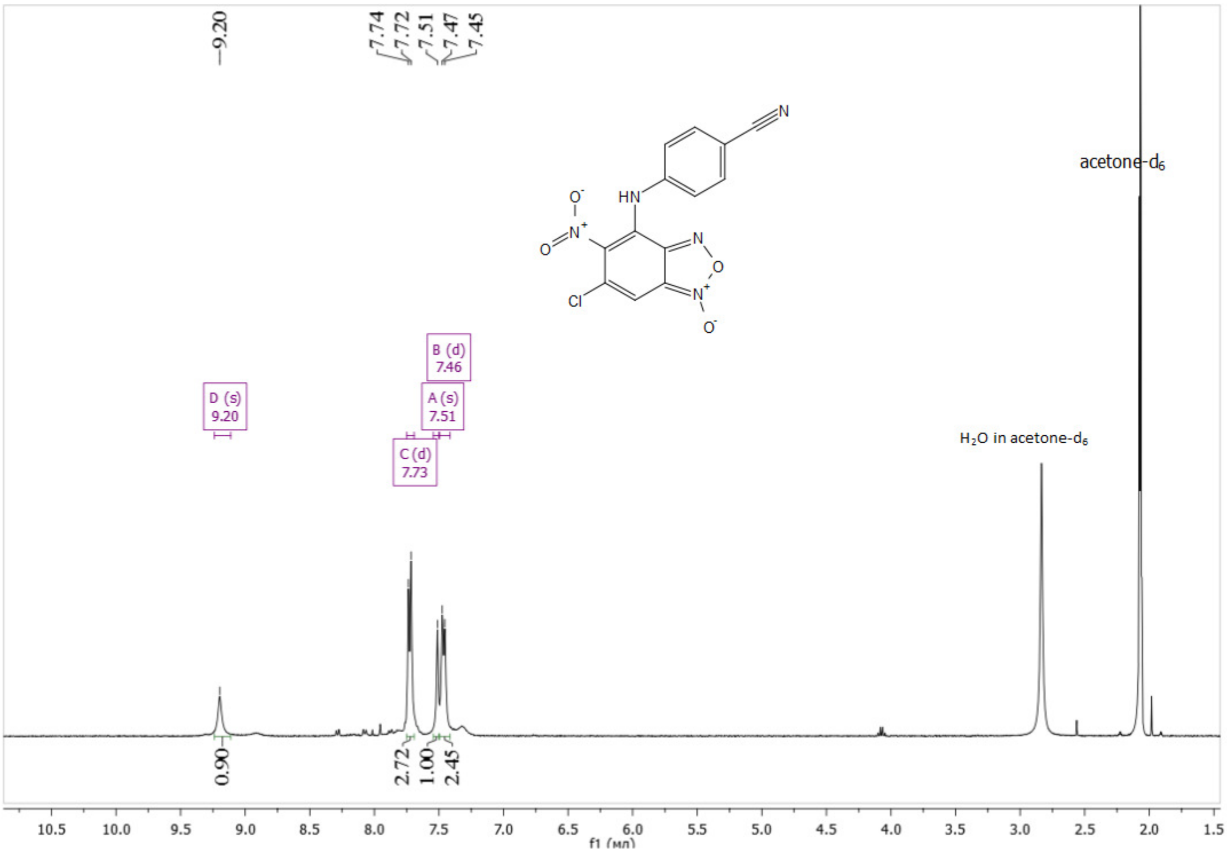

Figure S21. ^{1}H NMR (acetone-d_6, 400 MHz, 25 °C) of compound **3d**.

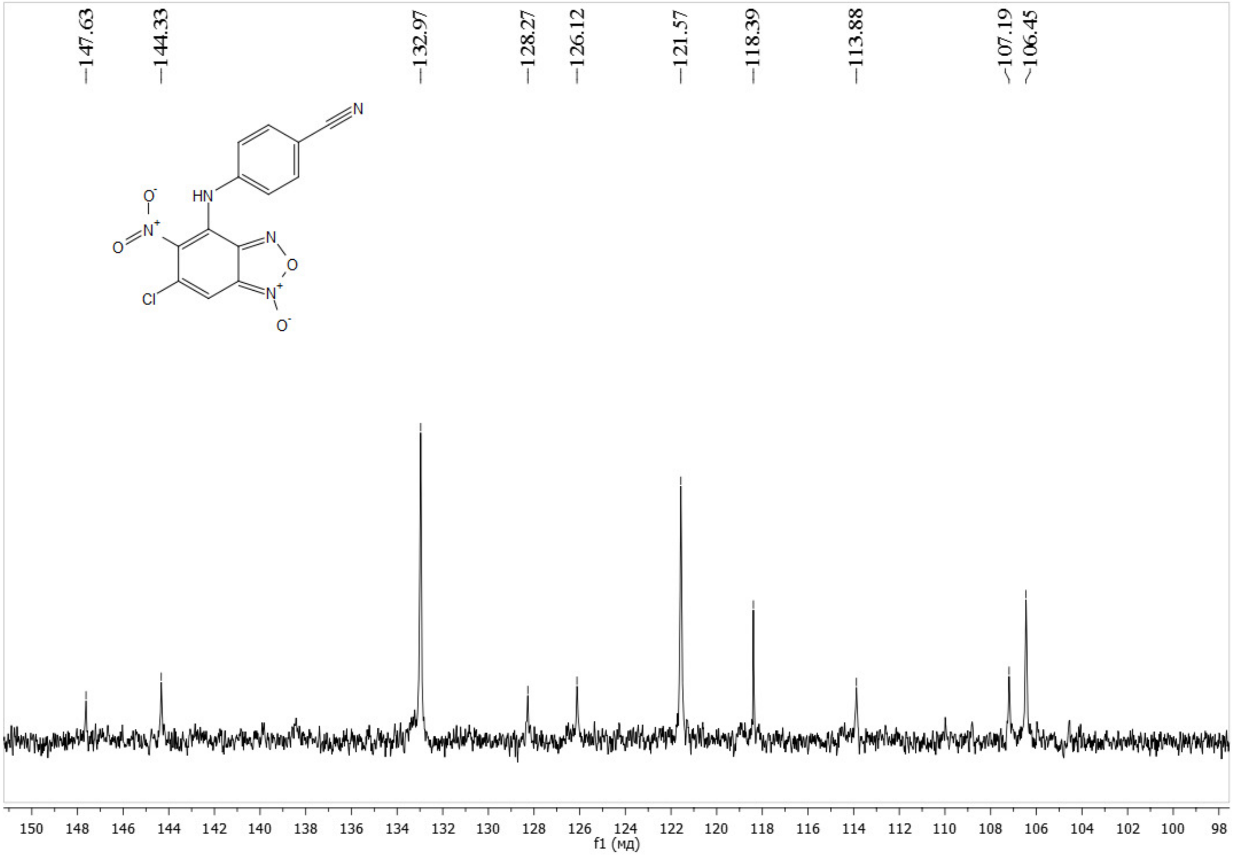

Figure S22. $^{13}C\{1H\}$ NMR (acetone-d_6, 151 MHz, 25 °C) of compound **3d**.

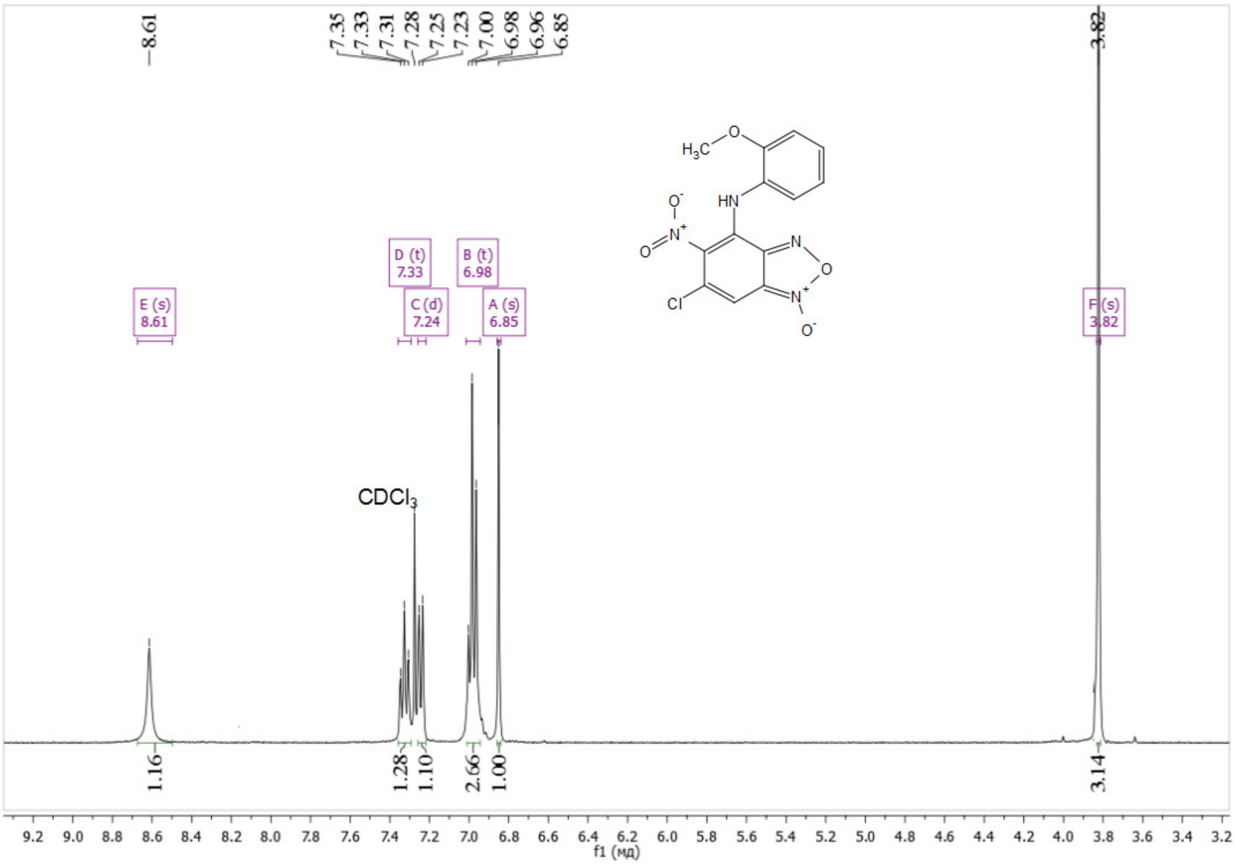

Figure S23. ^{1}H NMR ($CDCl_3$, 400 MHz, 25 °C) of compound **3e**.

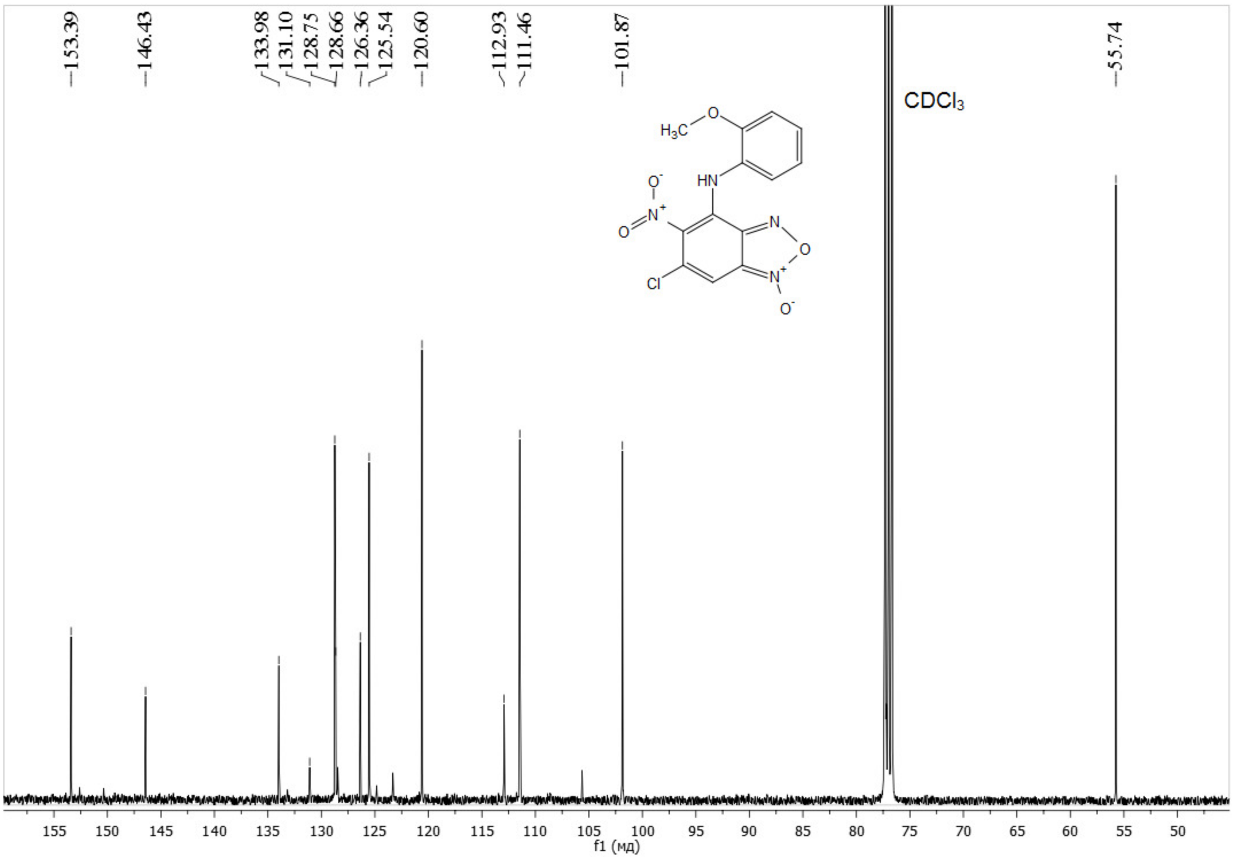

Figure S24. $^{13}C\{1H\}$ NMR ($CDCl_3$, 101 MHz, 25 °C) of compound **3e**.

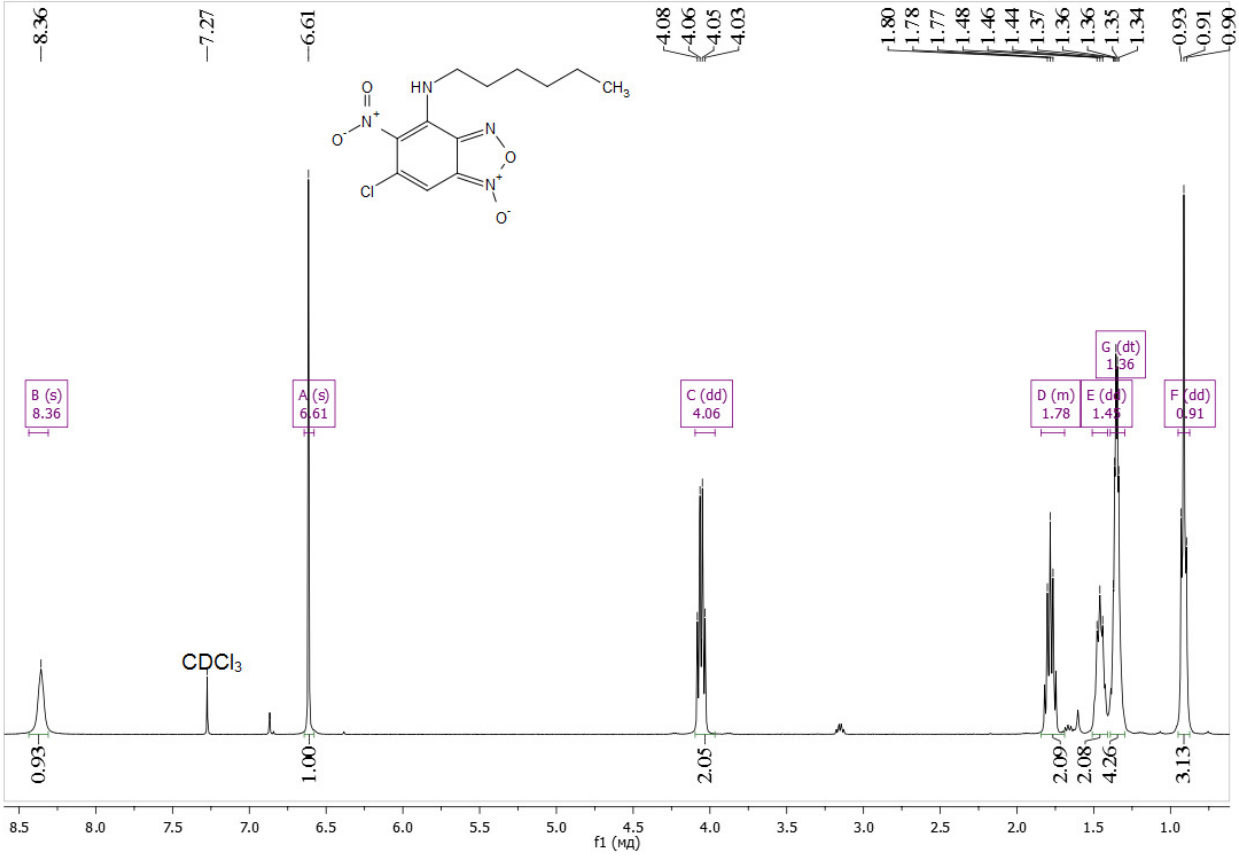

Figure S25. ^{1}H NMR ($CDCl_3$, 400 MHz, 25 °C) of compound **5b**.

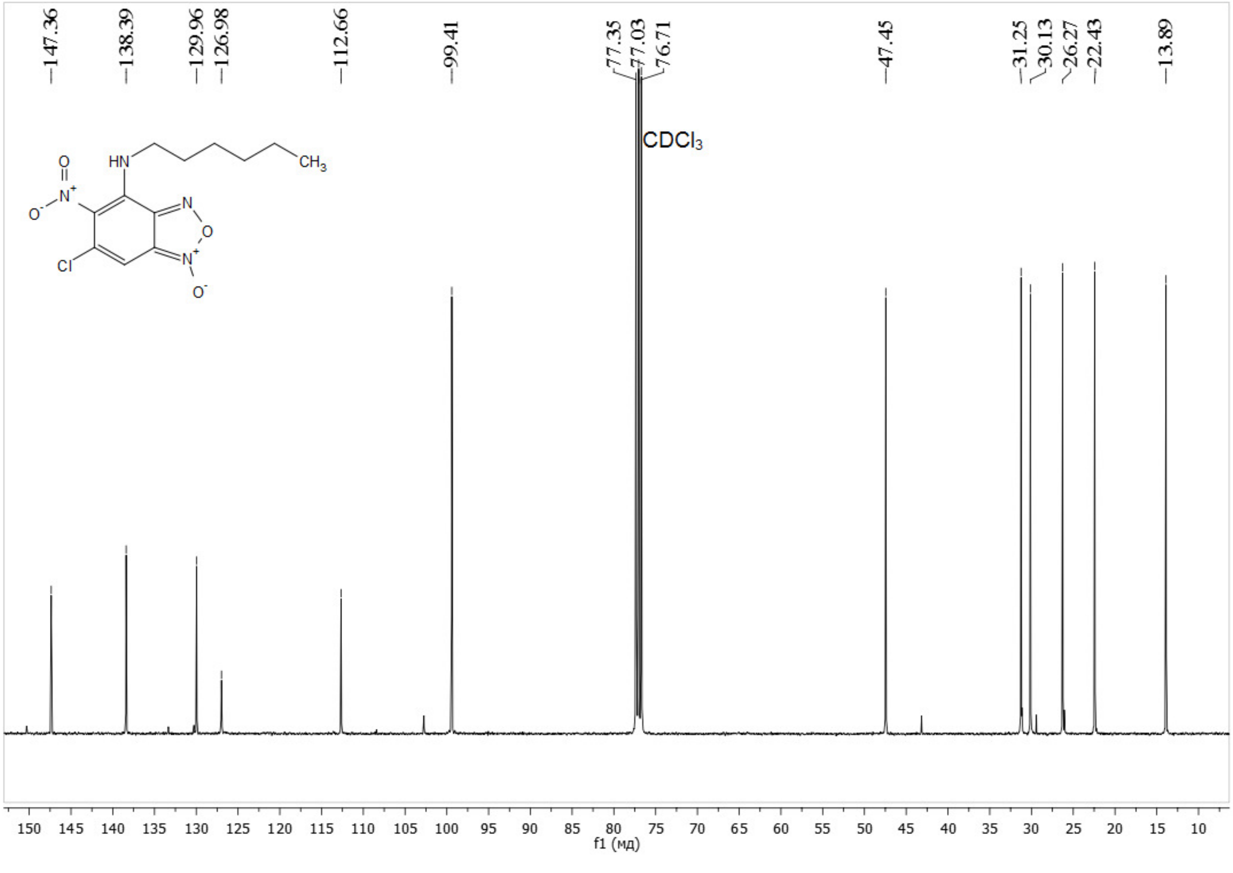

Figure S26. $^{13}C\{1H\}$ NMR ($CDCl_3$, 101 MHz, 25 °C) of compound **5b**.

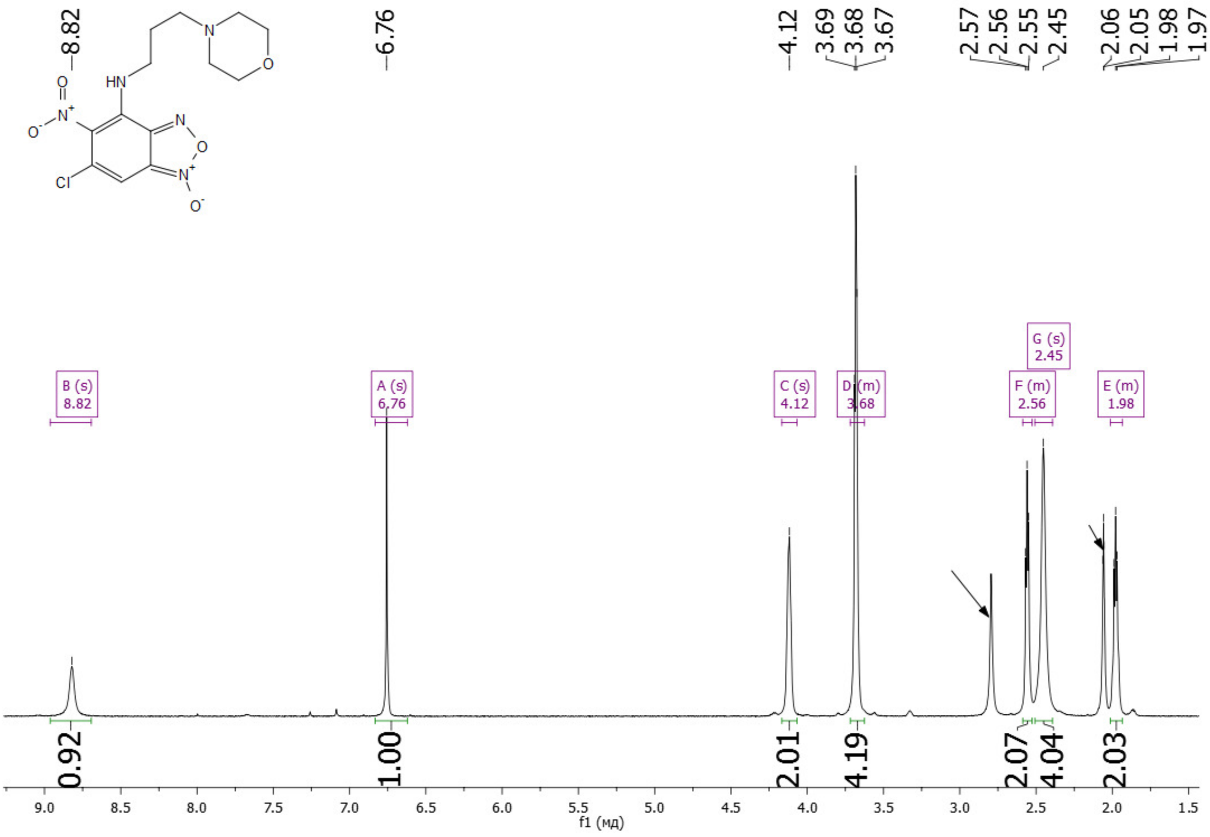

Figure S27. ^1H NMR (acetone-d$_6$, 600 MHz, 25 °C) of compound **5c**.

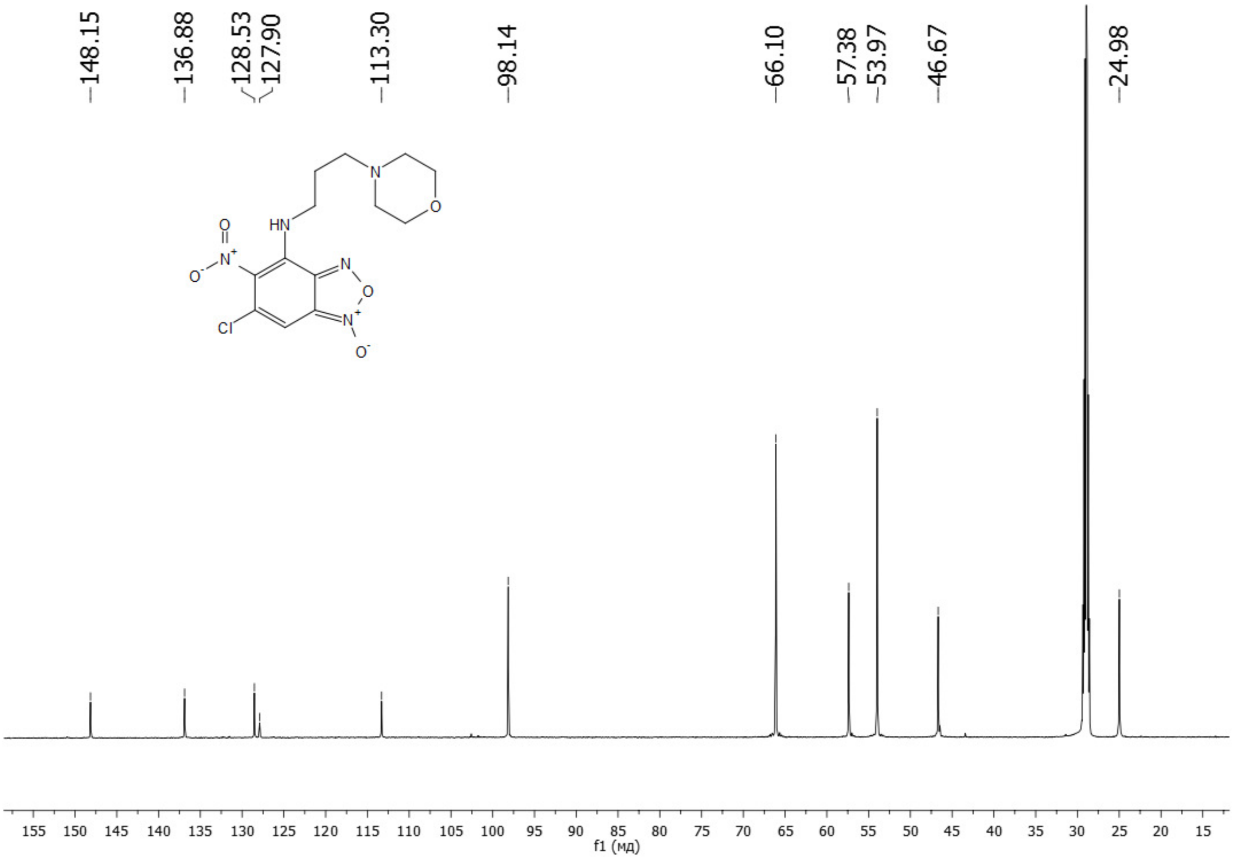

Figure S28. $^{13}C\{1H\}$ NMR (acetone-d$_6$, 151 MHz, 25 °C) of compound **5c**.

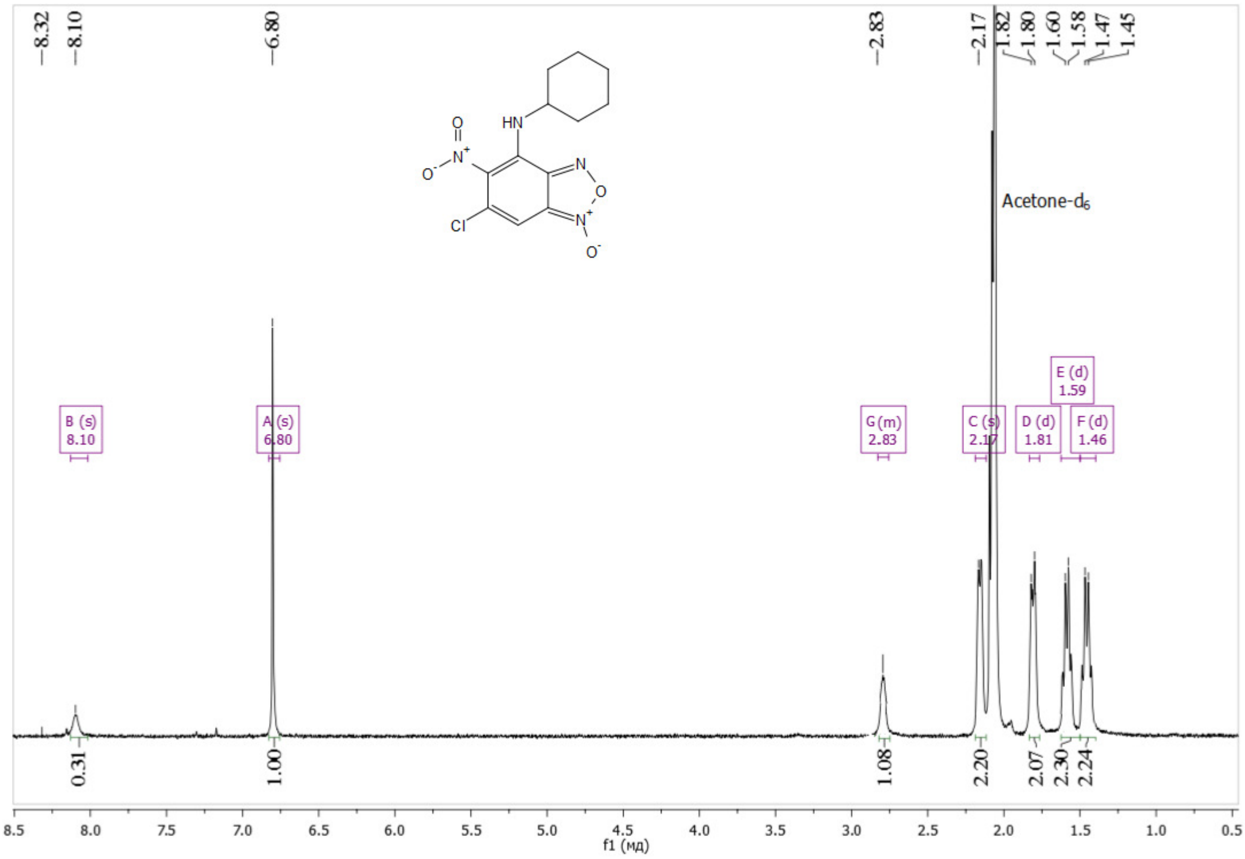

Figure S29. ^{1}H NMR (acetone-d$_6$, 600 MHz, 25 °C) of compound **5d**.

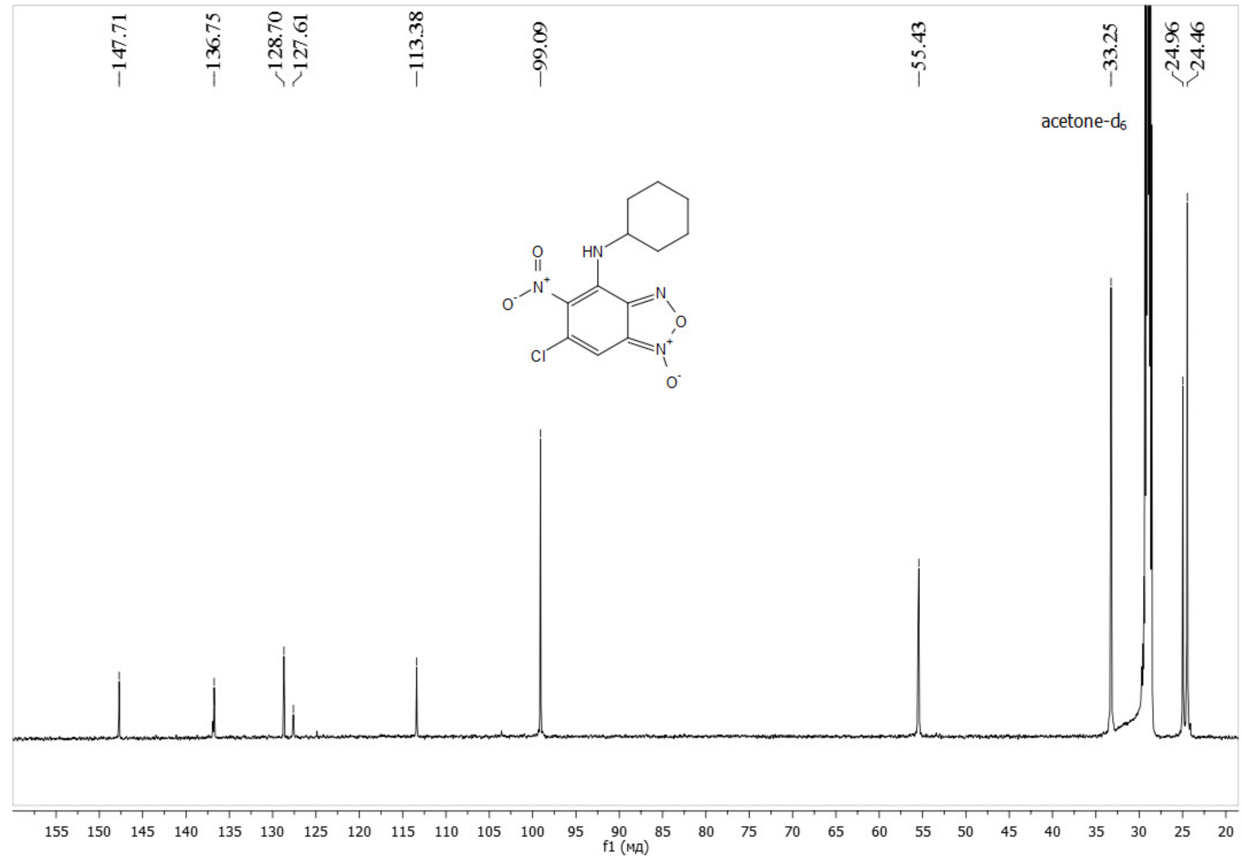

Figure S30. ^{13}C{1H} NMR (acetone-d$_6$, 151 MHz, 25 °C) of compound **5d**.

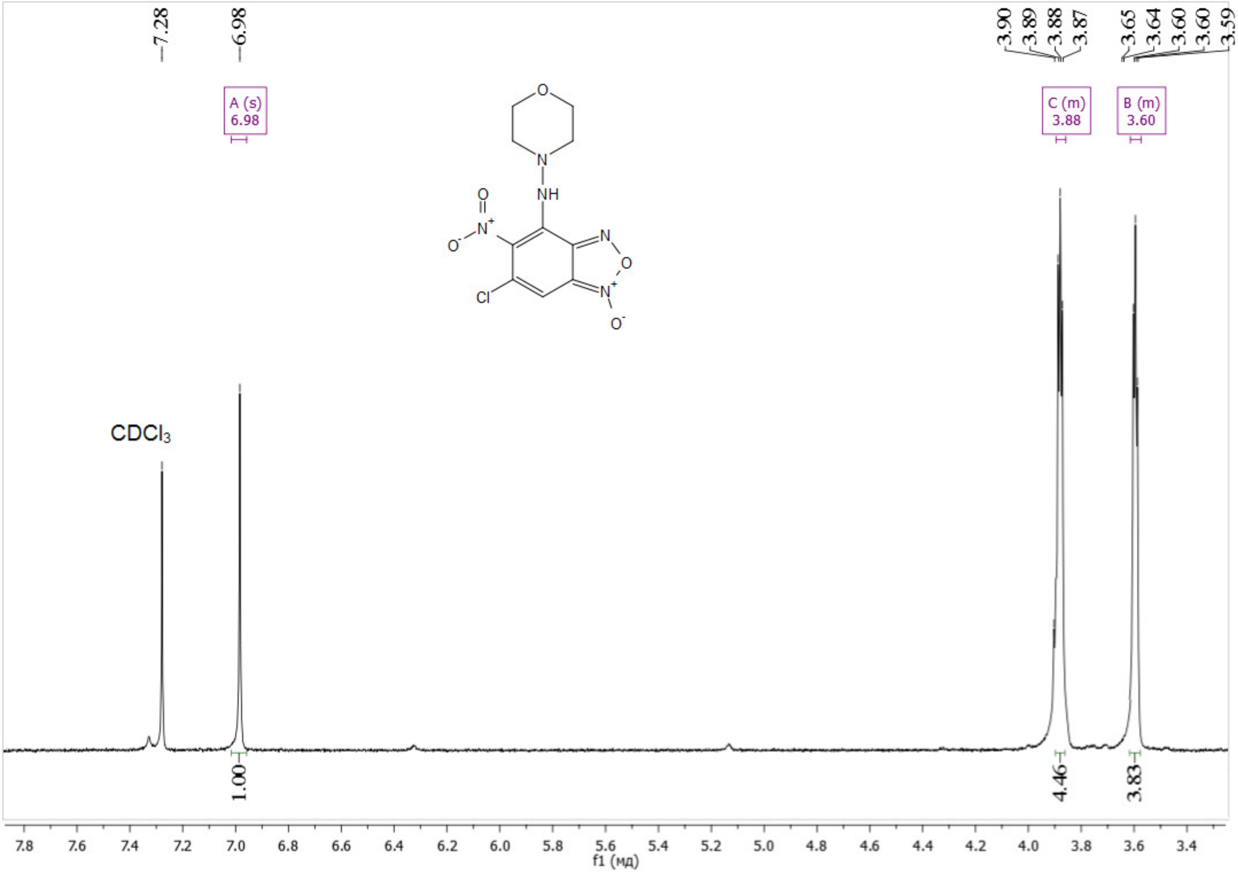

Figure S31. ^{1}H NMR ($CDCl_3$, 600 MHz, 25 °C) of compound **5e**.

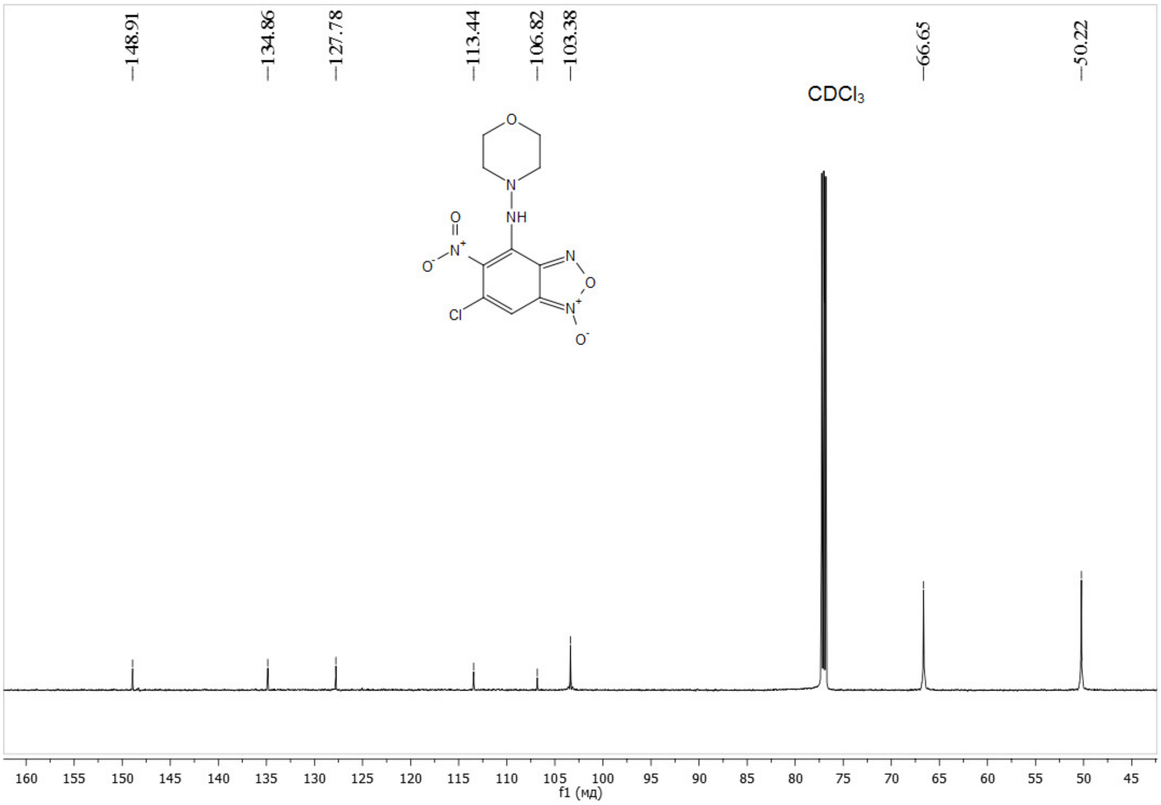

Figure S32. $^{13}C\{1H\}$ NMR ($CDCl_3$, 151 MHz, 25 °C) of compound **5e**.

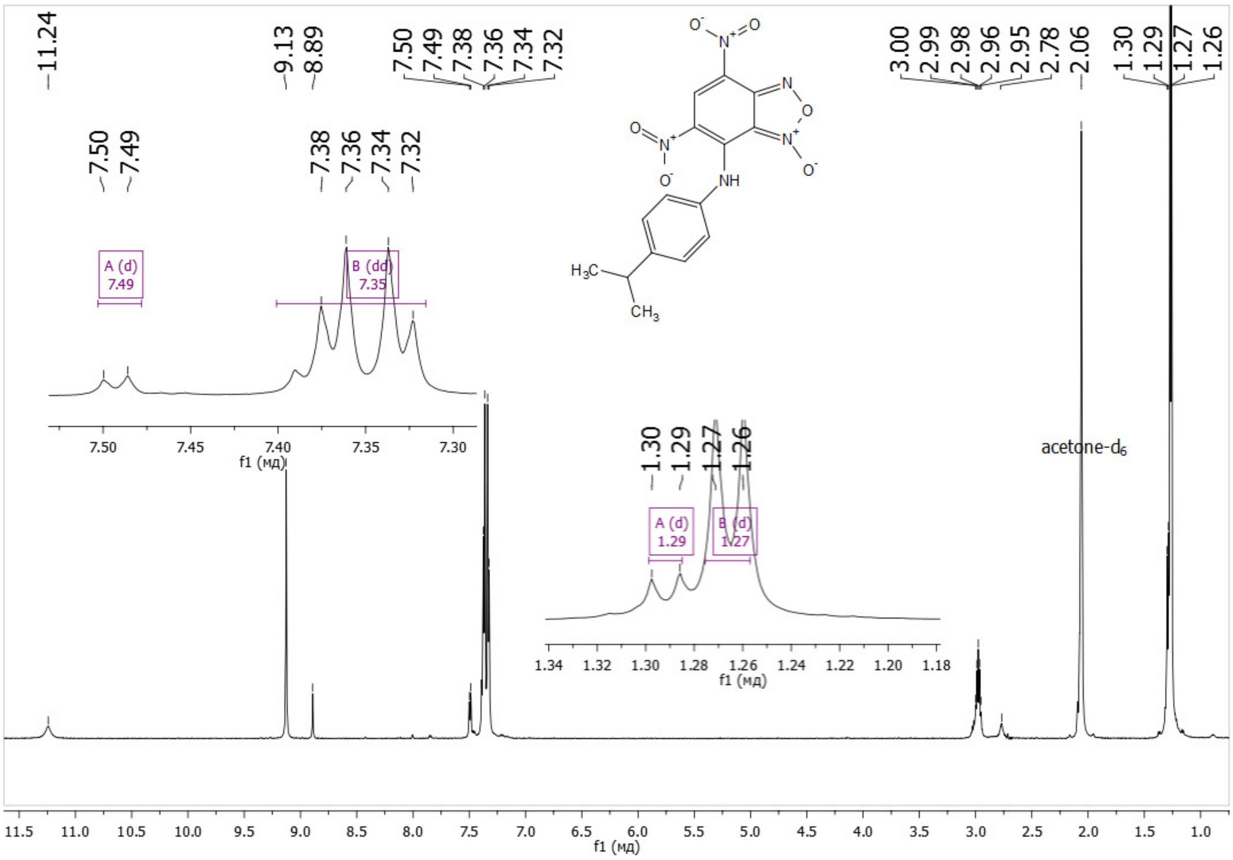

Figure S33. ^{1}H NMR (acetone-d_6, 600 MHz, 25 °C) of compound **6**.

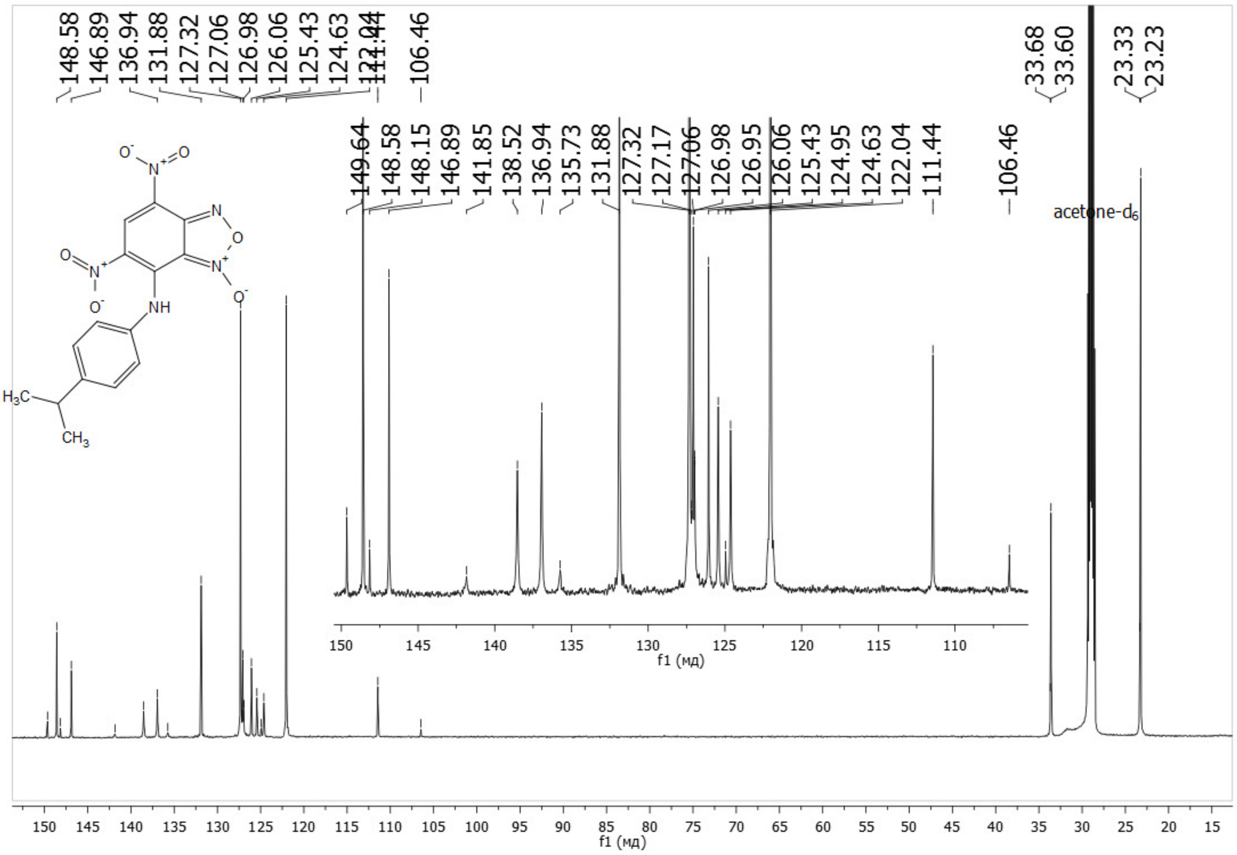

Figure S34. $^{13}C\{^{1}H\}$ NMR (acetone-d_6, 151 MHz, 25 °C) of compound **6**.
